# Endometriosis-related peritoneal adhesion formation: molecular mechanisms and clinical significance

**DOI:** 10.1093/humupd/dmag021

**Published:** 2026-08-14

**Authors:** Becky Macpherson, Joel Zindel, Michael Mueller, Erin Greaves

**Affiliations:** Warwick Medical School, University of Warwick, Coventry, UK; Department of Biology, Institute of Molecular Health Sciences, ETH Zurich, Zurich, Switzerland; Department of Obstetrics and Gynaecology, Inselspital Bern and University of Bern, Bern, Switzerland; Division of Biomedical Sciences, Centre for Early Life, Warwick Medical School, University of Warwick, Coventry, UK

**Keywords:** adhesions, peritoneal adhesions, endometriosis, peritoneum, fibrosis, macrophage, mesothelial cell, mesothelium

## Abstract

**BACKGROUND:**

Peritoneal adhesions are a major and often under-recognized cause of morbidity following abdominopelvic inflammation and surgery, contributing to chronic pelvic pain, infertility, bowel obstruction, and surgical complications. Endometriosis, affecting 10–11% of reproductive-age women, is strongly associated with adhesions; however, whether adhesion formation in endometriosis reflects general peritoneal injury or disease-specific mechanisms remains unclear.

**OBJECTIVE AND RATIONALE:**

This narrative review examines (i) physiological peritoneal healing and mechanisms of adhesion formation, (ii) how the altered peritoneal microenvironment in endometriosis may modify adhesiogenesis, and (iii) current and theoretical therapeutic and preventative strategies for adhesions in endometriosis. This will improve current understanding of adhesiogenesis and the implications of adhesions in women with endometriosis, and will identify areas for future research to reduce morbidity in this patient population.

**SEARCH METHODS:**

Literature searches were performed in PubMed, Embase and Cochrane Library using combinations of the terms ‘*endometriosis*’, ‘*adhesions*’, ‘*pelvic*’, ‘*abdominal*’, ‘*peritoneal*’ between January 2000 and February 2026. Randomized-controlled trials, observational studies, systematic reviews, and relevant experimental studies were included. Case reports and non-English publications were excluded. A total of 124 studies were included in this literature review.

**OUTCOMES:**

Adhesion formation results from dysregulated peritoneal repair involving inflammation, coagulation-fibrinolysis imbalance, and fibroproliferative remodelling. Evidence suggests that endometriosis creates a peritoneal environment characterized by chronic inflammation, altered immune responses, repeated tissue injury, and exposure to menstrual debris and iron, which may promote adhesiogenesis. However, much mechanistic understanding is derived from experimental models, and direct evidence from patients remains limited. Prevention of adhesiogenesis in endometriosis currently focuses on intra-operative interventions to limit adhesion development following endometriosis-related surgery, rather than preventing disease-related adhesiogenesis in the absence of surgery. We discuss potential pharmacotherapies, including hormonal treatments, identified by experimental studies, which may perturb endometriosis-related adhesiogenesis.

**WIDER IMPLICATIONS:**

Current data support a model in which adhesion biology in endometriosis largely reflects conserved wound-healing pathways but develop within a chronically altered peritoneal microenvironment that lowers the threshold for adhesiogenesis and promotes persistence and recurrence. Clarifying endometriosis-specific drivers and generating robust human data are essential to develop targeted adhesion-prevention strategies and improve long-term outcomes for affected patients. Notably, there are currently no effective pharmacological therapies to prevent or treat adhesions, representing a major unmet clinical need in endometriosis care.

**REGISTRATION NUMBER:**

N/A.

## Graphical Abstract

**Graphical Abstract:**
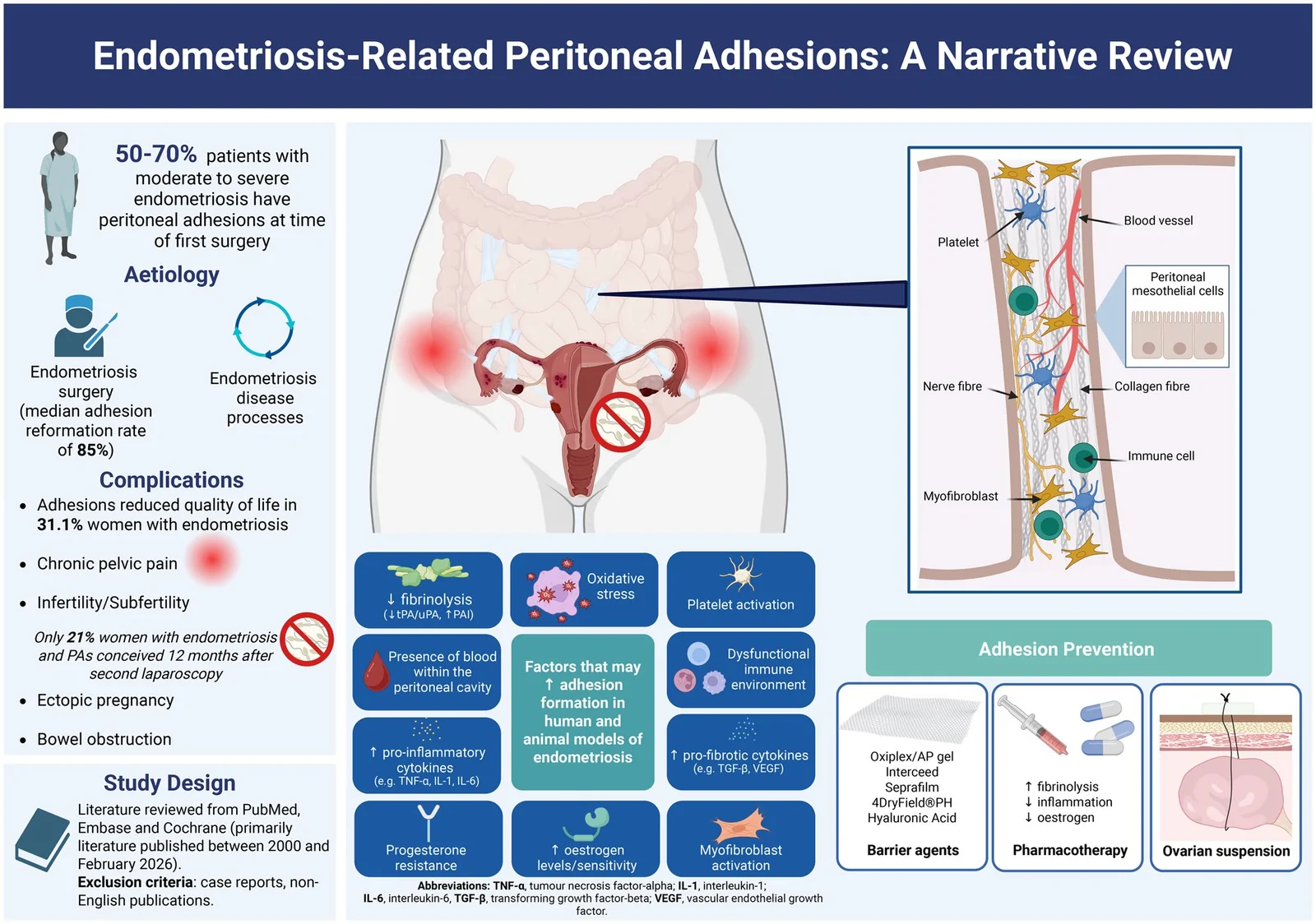
Endometriosis-Related Peritoneal Adhesion Formation: Molecular Mechanisms and Clinical Significance. Created in BioRender. Greaves, E. (2026) https://BioRender.com/pk1trxt

## Introduction

Peritoneal adhesions are among the most common and clinically significant sequelae of abdominopelvic injury and inflammation. They contribute to chronic abdominopelvic pain, infertility, bowel obstruction, and major complications during repeat surgery. Peritoneal adhesions are bands of connective tissue that develop between a site of peritoneal injury and another abdominopelvic surface. They occur following abdominopelvic injuries and other inflammatory challenges such as surgery, irradiation, and endometriosis (De Wilde *et al.*, 2016; Tabibian *et al.*, 2017). Independent of their cause, adhesions represent a significant health burden, causing complications including infertility, chronic pain, bowel obstruction, ectopic pregnancy, and ‘frozen pelvis’, whereby adhesions tether pelvic organs to adjacent structures (ten Broek *et al.*, 2013; Abd El-Kader *et al.*, 2019).

Endometriosis is a chronic inflammatory condition affecting approximately 11% of menstruating individuals, and is characterized by the implantation of endometrial-like tissue at extra-uterine locations, forming endometriotic ‘lesions’. Endometriosis often causes debilitating symptoms, including chronic abdominopelvic pain and subfertility/infertility (Ellis *et al.*, 2022). The pathobiology of endometriosis includes chronic peritoneal inflammation, immune dysregulation, and altered steroid receptor and steroidogenic enzyme expression (Ellis *et al.*, 2022). Sampson’s hypothesis of retrograde menstruation postulates that menstrual blood containing endometrial cells is refluxed into the peritoneal cavity through fallopian tubes, leading to implantation of endometrial-like cells at ectopic sites (Sampson, 1927) and is the most popular theory explaining endometriosis development. Paradoxically, historical reports suggest that up to 90% of menstruating individuals exhibit retrograde menstruation (although the prevalence of this was recently refuted (Viganò *et al.*, 2024)), emphasizing that survival of ectopic endometrial-like cells and their development into endometriotic lesions depends on additional factors that influence disease pathology (Halme *et al.*, 1984). Other theories include (i) the theory of mullerianosis, describing the misplacement of mullerian tissue during organogenesis, resulting in the establishment of endometriotic cells that may later form endometriotic lesions upon oestrogen stimulation (Batt *et al.*, 2007)), (ii) the theory of neonatal uterine bleeding (proposes that endometrial bleeding in the neonatal period allows fragments to enter the peritoneal cavity and develop as endometriotic lesions upon ovarian steroid activation (Brosens *et al.*, 2013)), and (iii) mesothelial transdifferentiation (Matsuzaki and Darcha, 2012).

Endometriotic lesions are commonly found on the pelvic peritoneum but are also found at lower frequencies in extrapelvic regions. Endometriotic lesions are composed of endometrial stromal cells, glandular endometrial epithelial cells, immune cells, and fibrotic areas. They are also vascularized and innervated (Vigano *et al.*, 2018; Saunders and Horne, 2021). Akin to eutopic endometrial cells, ectopic endometrial-like cells are functional; they proliferate in response to ovarian hormones, locally produce hormones, and studies suggest that some endometriotic lesions bleed during menstruation (Laux-Biehlmann *et al.*, 2015). A recent study, however, indicates that many endometriotic lesions are not synchronous with matched eutopic endometrium (Colgrave *et al.*, 2021).

Crucially, endometriotic lesions are associated with the presence of adhesions, most commonly involving the ovaries and the Pouch of Douglas (Somigliana *et al.*, 2012; Vercellini *et al.*, 2014). Adhesions can distort abdominopelvic anatomy, causing chronic pain, infertility, and intestinal obstruction. It is reported that patients with endometriosis-related adhesions had a lower quality of life relative to patients with endometriosis alone (De Wilde *et al.*, 2016; Abd El-Kader *et al.*, 2019). Importantly, adhesions are frequently present at the time of first surgery, i.e. in patients that never experienced surgical injury, supporting the concept that the endometriotic peritoneal niche is intrinsically pro-adhesiogenic. The mechanisms underlying this association will be explored in detail in this review.

The revised American Society for Reproductive Medicine (rASRM) staging criteria reflect the importance of adhesions in the endometriosis phenotype: the classifications are defined based on (1) endometriosis lesion size and localization, (2) extent of posterior cul de sac obliteration, and (3) severity of ovarian and tubal adhesions formed independent of endometriotic lesions by aberrant peritoneal repair (American Society for Reproductive Medicine, 1997).

Up to 50–70% of women with moderate-to-severe endometriosis have adhesions at the time of their first surgery (Abd El-Kader *et al.*, 2019), supporting the concept that the endometriotic niche is *sufficient* to cause adhesions in the absence of surgical history. However, when hormone-suppressing medication fails, surgery becomes an integral part of endometriosis management (Clark *et al.*, 2020). Symptom recurrence following surgery is a key issue, with 50–60% of patients opting for further surgery to manage symptoms within 5–7 years (Cheong *et al.*, 2008; Shakiba *et al.*, 2008; Stratton and Berkley, 2011). The incidence of post-operative adhesions after abdominopelvic surgeries is as high as 60–90% (Menzies and Ellis, 1990; Oboh and Trehan, 2007). Therefore, both endometriosis-induced adhesions and post-operative adhesions or the combination thereof, pose a significant burden to endometriosis patients and services. Although adhesions are traditionally viewed as a consequence of surgical injury, endometriosis patients frequently develop adhesions in the absence of acute trauma, suggesting that disease-alterations in peritoneal repair may predispose to pathological healing. A detailed comparison of post-surgical adhesiogenesis and endometriosis-associated peritoneal repair is therefore required to distinguish conserved mechanisms from disease-specific modifiers. In this review, we integrate current evidence to propose that adhesion formation in endometriosis reflects dysregulated activation of normal wound-healing pathways within a chronically altered peritoneal microenvironment, and we discuss the implications of this framework for understanding disease burden and improving preventative and therapeutic strategies.

## Methods

A structured literature search was conducted in PubMed, Embase and Cochrane Library using combinations of the terms ‘*endometriosis*’, ‘*adhesions*’, ‘*pelvic*’, ‘*abdominal*’, ‘*peritoneal*’. Titles and abstracts published between January 2000 and February 2026 were screened, with earlier landmark publications included to provide historical and mechanistic context. A total of 124 studies were included in this review. Reference lists of eligible articles were manually searched to identify additional relevant studies (Fig. 1).

**Figure 1.**
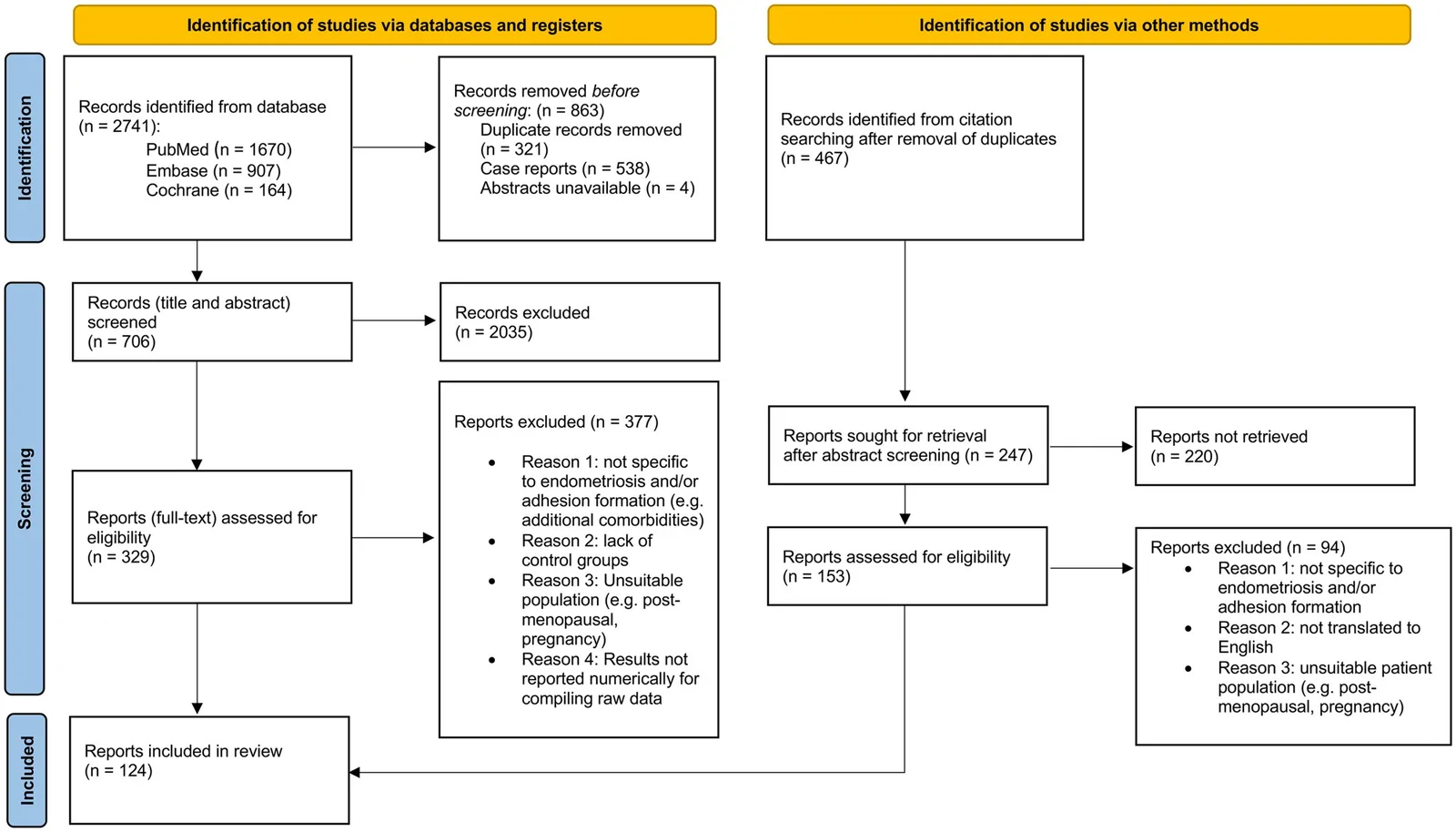
**Flow diagram showing the methodology of the literature review process**. Structured literature searches were conducted in PubMed, Embase, and Cochrane using combinations of the terms ‘*endometriosis*’, ‘*adhesions*’, ‘*pelvic*’, ‘*abdominal*’, and ‘*peritoneal*’ between the period January 2000 and February 2026. Additional relevant studies were identified by manual screening of reference lists of eligible articles. Adapted from Page *et al*. (2021).

Studies describing physiological peritoneal healing, mechanisms of adhesiogenesis, clinical associations between endometriosis and peritoneal adhesions, and strategies for prevention or treatment were included. Peer-reviewed randomised-controlled trials (RCT), meta-analyses, cohort studies, and systematic reviews were prioritized where available. Experimental animal studies and narrative reviews were included to inform mechanistic understanding where human data were limited. Case reports and non-English publications were excluded. Methodological quality of included studies was assessed using Joanne Briggs Institute critical appraisal tools (Joanna Briggs Institute, 2017).

Given the narrative nature of this review, formal meta-analysis was not performed; however, studies were critically evaluated with consideration of study design, translational relevance, and hierarchy of evidence.

## The structure and function of the peritoneum

The peritoneum is a continuous serous membrane composed of a monolayer of mesothelial cells overlaying a submesothelial connective tissue layer that encloses the abdominopelvic cavity and lines abdominal viscera. The peritoneum reduces intra-abdominal friction, provides structural and metabolic support to tissues, and serves as a protective barrier between the peritoneal cavity and abdominal wall, acting as a first line of defence against pathogens and metastatic cells. The peritoneum comprises a parietal layer, which lines the peritoneal surface of the abdominopelvic wall, and the visceral layer, which lines intraperitoneal tissues. The closed sac formed from the parietal and visceral membranes is known as the peritoneal cavity, containing 5–20 ml of peritoneal fluid. The peritoneum comprises the mesothelium, composed of a cobblestone-like monolayer of peritoneal mesothelial cells and the sub-mesothelium (Fig. 2) (Capobianco *et al.*, 2017; van Baal *et al.*, 2017).

**Figure 2.**
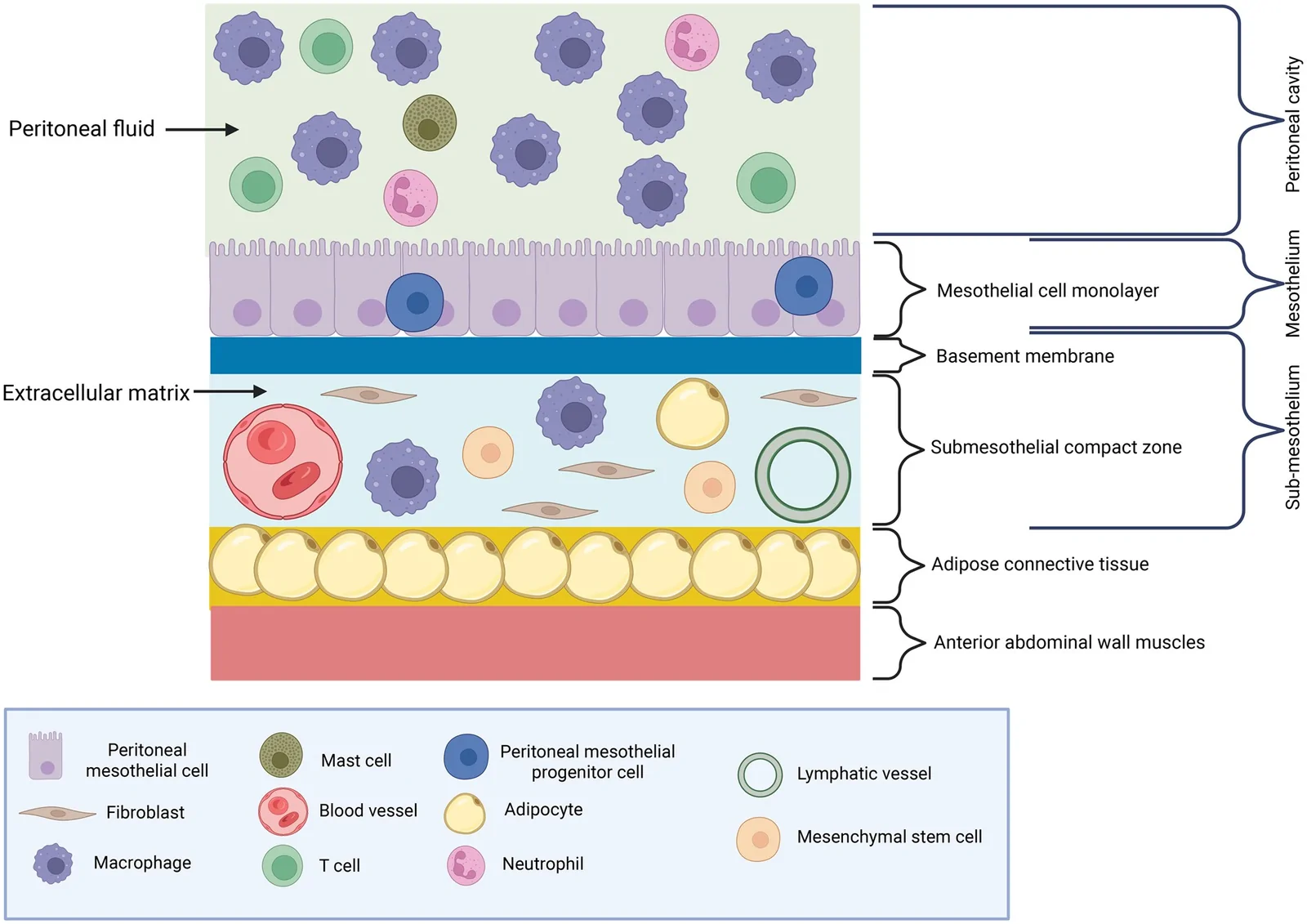
**Illustration of the structure of the peritoneum**. The peritoneum is composed of a monolayer of peritoneal mesothelial cells which lines the peritoneal cavity. Peritoneal mesothelial cells are polygonal-shaped cells with epithelial and mesodermal properties. The luminal surface of peritoneal mesothelial cells is lined with microvilli, which increase surface area for absorption, capture microorganisms, and protect the mesothelium against frictional injury (Chegini, 2002; Mutsaers *et al*., 1996). The sub-mesothelium is situated beneath the mesothelial cell monolayer and consists of the basement membrane and sub-mesothelial compact zone. The sub-mesothelial compact zone contains mesenchymal stem cells, adipocytes, fibroblasts, immune cells, connective tissue, and vasculature. The sub-mesothelial compact zone overlies adipose tissue and the anterior abdominal wall musculature (Capobianco *et al*., 2017). Created in BioRender. Greaves, E. (2026) https://BioRender.com/67tjjxc.

## Physiological peritoneal repair

Peritoneal repair shares many features with skin repair and involves overlapping phases: (**i) coagulation** (immediate to several hours), (**ii) inflammation** (Days 1–3), (**iii) proliferation** (Days 4–21) and (**iv) remodelling** (21 days to 1 year) (Hunt *et al.*, 2000; Landén *et al.*, 2016). Importantly, to structure the review, we will follow the paradigm and treat coagulation and inflammation as distinct paragraphs. However, the **interdependence and cooperative function of coagulation and inflammation,** also referred to as **thromboinflammation**, is now increasingly recognized in peritoneal repair.

### Coagulation (minutes to hours)

When the mesothelium is subjected to mechanical stress or microbial damage, inflammatory and coagulation cascades are activated, causing fibrin clot formation that limits blood loss and supports repair (Shapiro *et al.*, 2015; Capobianco *et al.*, 2017). This pro-coagulant response is normally counterbalanced by fibrinolysis, which restricts fibrin deposition to the site of injury and prevents excessive or persistent fibrin accumulation and fibrocollagenous tissue accumulation and adhesiogenesis at non-injured sites, so the fibrin deposit is restricted to injured areas to permit peritoneal mesothelial cell replacement (re-mesothelialization) as well as repair of the submesothelial connective tissue. Following peritoneal injury, a tightly regulated balance between coagulation, anticoagulation, inflammation, and fibrinolysis normally results in fibrin clearance within ∼72 h (Arung *et al.*, 2011; Koninckx *et al.*, 2016; Rabieian *et al.*, 2018).

Detection of injury by macrophages and intraperitoneal bleeding leads to the activation of coagulation pathways, promoting fibrin deposition and further immune cell recruitment (Haney, 2000; Cheong *et al.*, 2001). Furthermore, activated mast cells, which are also abundant under homeostatic conditions, release pro-inflammatory cytokines and vasoactive components, causing vasodilation and extravasation of coagulation components, which further induces the coagulatory cascade (Cahill *et al.*, 2006; Capobianco *et al.*, 2017).

Importantly, pro-coagulation factors such as tissue factor do not only come from the circulatory system (endothelium, platelets, liver) (Kothari *et al.*, 2009) but are also expressed directly by surrounding mesothelial cells and resident peritoneal macrophages (Capella-Monsonís *et al.*, 2019; Zhang *et al.*, 2019a). The release of tissue factor results in the activation of the coagulation cascade, which culminates in the activation of thrombin. Thrombin catalyzes fibrinogen activation to fibrin and is inhibited by antithrombin III. Fibrin is deposited at sites of mesothelial injury and is subsequently stabilized by Factor XIIIa-mediated cross-linking, forming a provisional matrix. Platelets may continue this process when peritoneal injury is accompanied by microvascular bleeding and have also been implicated in endometriosis-associated peritoneal inflammation (Chegini, 2002; Sulaiman *et al.*, 2002; Kothari *et al.*, 2009; Hellebrekers and Kooistra, 2011; Luyendyk *et al.*, 2025).

A delicate balance exists between two processes occurring in parallel: coagulation pathway activation and subsequent fibrin generation at injured mesothelium, and the removal of fibrin by fibrinolysis. In fibrinolysis, plasmin (an enzyme generated by plasminogen cleavage) is responsible for fibrin degradation. Plasminogen is activated by tissue plasminogen activator (tPA), expressed by peritoneal mesothelial cells, macrophages, and endothelial cells, and urokinase plasminogen activator (uPA). Under homeostatic conditions, this system is strongly tuned towards fibrinolysis to enable rapid fibrin clearance and injury resolution. However, during thromboinflammation, this balance shifts toward fibrin persistence, facilitated by plasminogen activator inhibitors (PAI-1/2); glycoproteins expressed by peritoneal mesothelial cells, endothelial cells, macrophages, and fibroblasts that counteract tPA/uPA. During thromboinflammation, fibrinolysis (high tPA/uPA, low PAI-1/2) exceeds fibrinogenesis (low tPA/uPA, high PAI-1/2), and only once the insult resolves can fibrinolytic dominance be re-established to prohibit adhesiogenesis (Koninckx *et al.*, 2016; Honjo *et al.*, 2017; Capella-Monsonís *et al.*, 2019).

Furthermore, peritoneal mesothelial cells modulate degradation of extracellular matrix deposits on fibrin scaffolds by synthesizing matrix metalloproteinases (MMPs) and their inhibitors, tissue inhibitors of MMPs (TIMPs) (Chegini *et al.*, 2001). Strict regulation of this balance between extracellular matrix synthesis and degradation is also of key importance in attenuating adhesiogenesis in normal homeostasis. In summary, synthesis and degradation of fibrin occur in parallel, and a balance towards fibrinolysis, and the removal of any deposited extracellular matrix proteins on established fibrin scaffolds, is critical in preventing adhesiogenesis.

### Inflammation (minutes to days)

The innate immune system responds to damage-associated molecular patterns (DAMPs) or pathogen-associated molecular patterns released by injured peritoneal tissues (Zwicky *et al.*, 2021). Leukocyte infiltration is triggered by chemokine release from mesothelial cells, endothelial cells, and peritoneal resident macrophages, and is facilitated by adhesion molecules and integrins expressed by peritoneal resident macrophages (Mutsaers, 2002; Wang *et al.*, 2008; Speyer and Ward, 2011).

Peritoneal macrophages have emerged as key regulators of peritoneal homeostasis and repair. Peritoneal resident macrophages comprise approximately 50% of immune cells within the healthy peritoneal cavity (Haney, 2000; Guo *et al.*, 2020) and are crucial for pathogen defence, tumour cell clearance, and self-tolerance (Panir *et al.*, 2022). Observational studies demonstrate that macrophages accumulate in peritoneal fluid following abdominal surgery and inflammatory conditions, supporting their role in coordinating local immune responses and tissue repair within the peritoneal cavity (Bain and Jenkins, 2018; Guo *et al.*, 2020; Louwe *et al.*, 2021; Vega-Pérez *et al.*, 2021; Jayakumar *et al.*, 2022; Sahputra *et al.*, 2022; Liu *et al.*, 2025). However, much of the mechanistic understanding of peritoneal macrophage biology and their role in peritoneal repair derives from experimental animal models, particularly mice, and direct confirmation in human peritoneal healing remains limited.

In murine models, peritoneal resident macrophages are categorized by expression of the transcription factor GATA6^+^ (Okabe and Medzhitov, 2014; Rosas *et al.*, 2014) and because of their size, they have also been referred to as large peritoneal macrophages (LpMs). LpMs comprise 90% of peritoneal resident macrophages, and GATA6^-^ small peritoneal macrophages (SpMs) represent approximately 10% of peritoneal resident macrophages under steady state conditions (Ghosn *et al.*, 2010). Although LpMs predominate, their numbers rapidly diminish in response to inflammation in a process called the ‘macrophage disappearance reaction’ (MDR). It is postulated that in response to injury, rapid macrophage aggregation is responsible for this drop in cavity resident LpM. Several theories regarding the fate of LpMs during the MDR have been postulated, including drainage to omentum or lymph nodes, cell death or migration and adhesion to injured sites (Barth *et al.*, 1995; Okabe and Medzhitov, 2014). In all cases, MDR signifies the detection of a deviation from homeostasis by peritoneal immunity. In contrast, SpMs are monocyte-derived macrophages that rapidly increase in abundance during peritoneal inflammation and transiently become the principal macrophage type within the peritoneal cavity (Jayakumar *et al.*, 2022). Converting cavity macrophages are an intermediate Lyve1+ macrophage subset that arise from the differentiation of SpMs into LpMs, and retain functional differences to monocyte-derived and resident macrophages (Louwe *et al.*, 2021).

In humans, peritoneal resident macrophage populations were classified into three subsets according to expression of CD14/CD16 (CD14^++^CD16^+^, CD14^++^CD16^−^, and CD14^HI^CD16^HI^). The CD14^HI^CD16^HI^ subset was reported to express transcription factor GATA6 and was thus postulated to represent the mature peritoneal macrophage subset under steady-state conditions (Ruiz-Alcaraz *et al.*, 2018). However, a recent study reported that GATA6 expression in human peritoneal macrophages is very rare and weak in humans. The authors postulated that, instead the majority of human peritoneal macrophages exhibit a phenotype akin to converting cavity macrophages, a differentiation state just preceding expression of GATA6 (Han *et al.*, 2024). These findings highlight important interspecies differences and underscore the need for further investigation of human peritoneal macrophage biology.

Experimental studies have provided insight into how macrophages contribute to early peritoneal repair. Following injury, macrophages rapidly migrate to the damaged site and adhere to exposed extracellular matrix and to each other, forming an aggregate cell barrier over injured mesothelium. In mouse models, peritoneal macrophages form a barrier covering approximately 60% of injured mesothelium 15 min to 3 h following injury (Wang and Kubes, 2016; Ito *et al.*, 2021; Zindel *et al.*, 2021b). Within 5 days post-injury, the macrophage barrier was replaced by a new mesothelial lining (Cheong *et al.*, 2001; Ito *et al.*, 2021). When macrophages were unable to completely enclose the fibrin clot at injured mesothelium, adhesion formation ensued (Ito *et al.*, 2021; Zindel *et al.*, 2021b). Therefore, the macrophage barrier may be crucial for preventing adhesiogenesis. Furthermore, macrophages may promote mesothelial repair through their fibrinolytic activity (Burnett *et al.*, 2006) and production of transforming growth factor-beta (TGF-β) and other mediators involved in early healing (Cheong *et al.*, 2001; Landén *et al.*, 2016). However, the effects of macrophages on adhesion formation appear to depend on injury context and macrophage subset involved. For example, in some post-surgical adhesion models, depletion of peritoneal cavity macrophages using clodronate liposomes or Csflr-iDTR-based strategies reduced adhesion formation (Honjo *et al.*, 2017; Zindel *et al.*, 2021b).

Following macrophage aggregation, neutrophils infiltrate injured peritoneum and drive inflammation by releasing pro-inflammatory reactive oxygen species (ROS) and proteases (Vural *et al.*, 1999). Neutrophils also form neutrophil extracellular traps by ‘NETosis’, which promote platelet activity and coagulation, and resolution of inflammation (Tsai *et al.*, 2019; Elrod *et al.*, 2023).

### Proliferation and tissue remodelling

At most epithelial surfaces, wound healing occurs through gradual replacement of damaged epithelium by cells migrating and proliferating from the wound edges (Rodrigues *et al.*, 2019). In contrast, peritoneal repair appears to involve additional mechanisms that enable rapid restoration of mesothelial integrity. Following injury, peritoneal mesothelial cells can form discrete ‘islands’ across the injured surface, which subsequently expand and interconnect to reestablish a continuous mesothelial layer within 5–6 days (Hubbard *et al.*, 1967; Shapiro *et al.*, 2015; Tang *et al.*, 2020). Furthermore, peritoneal mesothelial cells may detach from the basement membrane and release DAMPs which promote inflammatory signalling and tissue repair responses (Yáñez-Mó *et al.*, 2003; Capobianco *et al.*, 2017).

The cellular derivation of peritoneal mesothelial cells involved in re-mesothelialization remains controversial. Experimental murine studies suggest that proliferation of peritoneal mesothelial cells at intact regions adjacent to injured mesothelium represents a major mechanism of repair, with cell division occurring 24–48 h after injury in response to macrophage-derived mediators (Mutsaers *et al.*, 2000). Supporting additional mechanisms, a study using cultured human mesothelial cells in a rat model of peritoneal injury detected free-floating mesothelial cells within peritoneal fluid that migrated to injured sites and contributed to mesothelial restoration (Foley-Comer *et al.*, 2002). Alternative cellular sources have also been proposed. Mesothelial cells participating in repair may arise from sub-mesothelial progenitor populations or through metaplastic conversion of sub-serosal fibroblasts underlying injured mesothelium (Raftery, 1973; Yung and Davies, 1998; Mutsaers *et al.*, 2000). However, experimental injury models in mice demonstrate fewer proliferating sub-mesothelial cells than dividing surface peritoneal mesothelial cells (Mutsaers *et al.*, 2000), suggesting this compartment contributes only modestly to mesothelial regeneration. Circulating progenitors may also participate, and bone marrow-derived cells expressing the mesothelial marker mesothelin have been shown to adhere to injured peritoneum in murine models (Carmona *et al.*, 2011). Therefore, mesothelial cells from multiple origins may contribute to re-mesothelialization following peritoneal injury. Together, coordinated regulation of coagulation-fibrinolysis, inflammatory resolution, immune cell function, and mesothelial regeneration ensures restoration of peritoneal integrity following injury. Disruption of these tightly regulated processes underlies pathological repair and adhesiogenesis, discussed in the following section.

## Adhesion formation: pathological peritoneal repair

Under certain circumstances, the ideal scenario of physiological peritoneal repair fails or is insufficient, leading to an alternative form of repair associated with the formation of excessive ectopic scars called adhesions.

The core mechanisms underlying this pathological form of repair (adhesion formation) include impaired fibrinolysis, altered extracellular matrix turnover (Coccolini *et al.*, 2013; Foster *et al.*, 2020), and sustained release of pro-inflammatory, pro-fibrotic cytokines and growth factors (Chegini, 2002). Peritoneal hypoxia also contributes to promoting fibrosis and neovascularization (Saed and Diamond, 2002; Fletcher *et al.*, 2008). We have discussed the importance of fibrin for repair and how coagulation is tightly linked to inflammation. In this scenario, fibrin persists on the basement membrane for approximately 24 h before degradation (Suzuki *et al.*, 2015). However, under sustained thromboinflammation, fibrinolysis is downregulated, and fibrin deposition exceeds fibrinolytic clearance, resulting in an overshooting repair scaffold. While this fibrin clot is required for physiological repair as a scaffold for extracellular matrix deposition by myofibroblasts (Capella-Monsonís *et al.*, 2019), the formation of overshooting fibrin bands allows attachment to adjacent peritoneal surfaces and ultimately irreversible ectopic scar tissue (Fig. 3).

**Figure 3.**
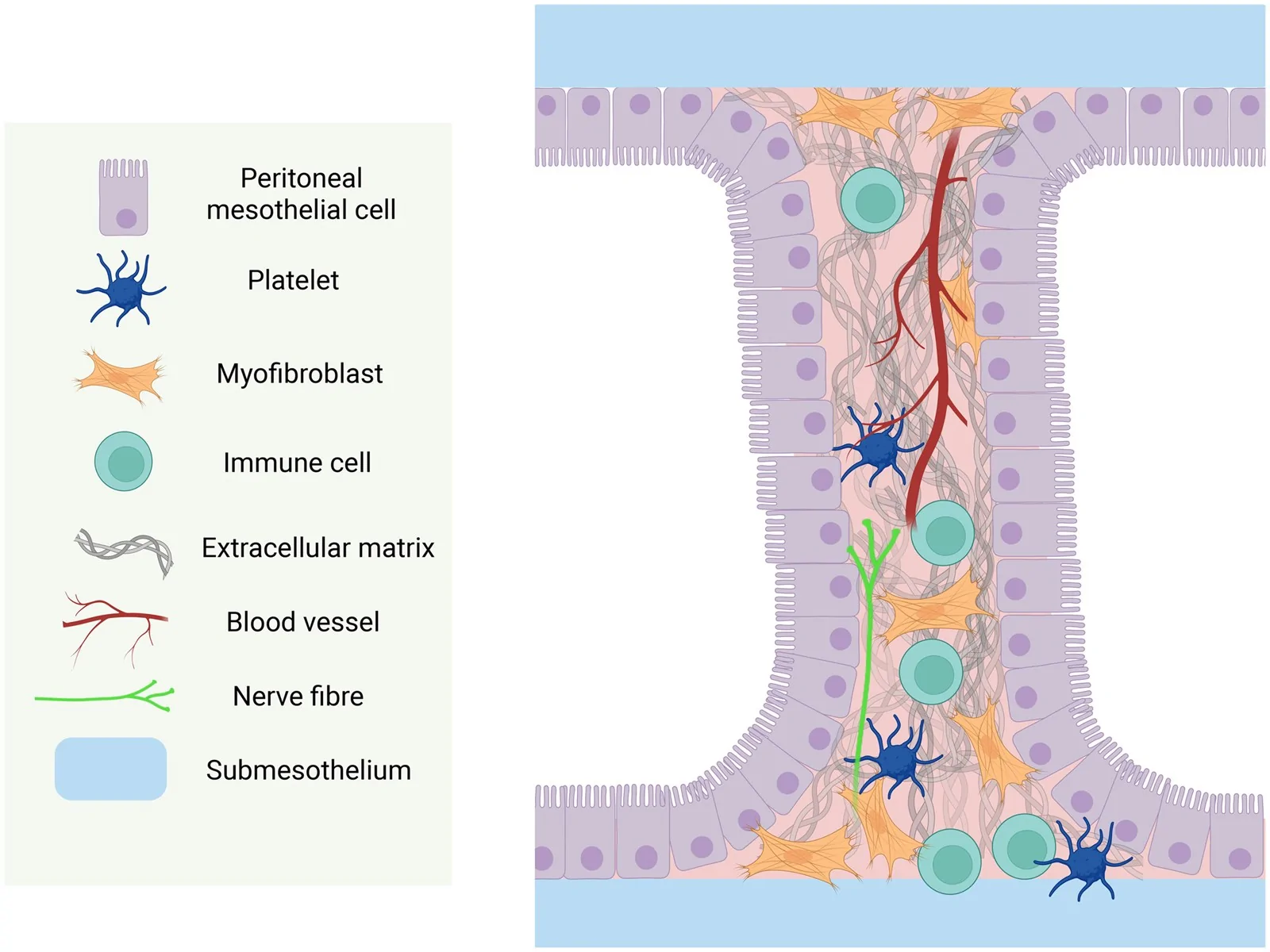
**The structure of an established peritoneal adhesion**. A peritoneal adhesion is composed of an interconnected network of extracellular matrix molecules (predominantly collagen) lined by peritoneal mesothelial cells. A diverse population of cells is involved in tissue remodelling, including immune cells, myofibroblasts and platelets. Peritoneal adhesions also develop nerve fibres and blood vessels. (Tabibian *et al*. 2017; Capella-Monsonís *et al*. 2019). Created in BioRender. Greaves, E. (2026) https://BioRender.com/2b8c7g4.

### The role of myofibroblasts in adhesion formation

Fibroblasts are spindle-shaped mesenchymal cells responsible for extracellular matrix maintenance and remodelling (Foster *et al.*, 2020). Upon peritoneal injury, differentiation into adhesion-specific myofibroblasts is promoted by pro-fibrotic growth factors and cytokines, including TGF-β1, and by increased extracellular matrix stiffness (Micallef *et al.*, 2012; Padwal and Margetts, 2016). Adhesions contain abundant alpha-smooth muscle actin (α-SMA)^+^ platelet-derived growth factor receptor (PDGFR)+ myofibroblasts, contractile cells that are largely absent from the homeostatic peritoneum (Micallef *et al.*, 2012). Adhesion myofibroblasts exhibit altered expression of type 1 collagen (Col1a1), MMP-1, PAI-1, tPA, TIMP-1, cyclooxygenase-2, and TGF-β, together with reduced fibrinolytic activity, consistent with their activated, matrix-producing phenotype that promotes extracellular matrix accumulation and adhesion maturation (Saed and Diamond, 2003; Rout *et al.*, 2005; Alpay *et al.*, 2008; Saed *et al.*, 2008; Ambler *et al.*, 2012b; Awonuga *et al.*, 2022).

The peritoneal fibroblast population is highly heterogeneous in both origin and functionality (Foster *et al.*, 2020), and the cellular source of adhesion-specific (myo)fibroblasts remains debated. Multiple progenitor populations have been proposed, including submesothelial fibroblasts and mesothelial cells undergoing mesothelial-to-mesenchymal transition (MMT), a type of epithelial-to-mesenchymal transition (EMT) occurring in chronic inflammation whereby epithelial markers including E-cadherin are downregulated and peritoneal mesothelial cells acquire mesenchymal markers including α-SMA, vimentin and Col1a1 (Foley-Comer *et al.*, 2002; Yáñez-Mó *et al.*, 2003; Loureiro *et al.*, 2013; Sandoval *et al.*, 2016; Zhang *et al.*, 2016a; Foster *et al.*, 2020). In the MMT explanatory model, myofibroblasts are derived from the mesothelium of adjacent or opposing peritoneal surfaces (Yáñez-Mó *et al.*, 2003; Chen *et al.*, 2014; Sandoval *et al.*, 2016; Uyama *et al.*, 2019; Foster *et al.*, 2020). In contrast, other authors suggested that submesothelial fibroblasts represent a major contributor to myofibroblast accumulation following injury (Chen *et al.*, 2014; Foster *et al.*, 2020). For example, fate-mapping approaches using Col1a1 lineage tracing demonstrated substantial contributions from submesothelial fibroblasts, but this was in response to a chemically induced peritoneal fibrosis, a biologically distinct process characterized by diffuse matrix deposition rather than focal adhesion formation (Chen *et al.*, 2014). A lineage-tracing study reported that myofibroblasts arising during adhesiogenesis originate from pre-existing PDGFR^+^ fibroblasts (Foster *et al.*, 2020). However, fate labelling in that study was induced perioperatively, and mesothelial cells undergoing MMT rapidly acquire PDGFR expression, meaning that newly transitioning mesothelial cells may also have been labelled. Conversely, increasing evidence supports a role for mesothelial plasticity in generating myofibroblasts through MMT, a process first proposed by Yáñez-Mó, López-Cabrera and colleagues and subsequently demonstrated across multiple experimental systems (Yáñez-Mó *et al.*, 2003; Aguilera *et al.*, 2005; Sandoval *et al.*, 2016; Fischer *et al.*, 2020; Zindel *et al.*, 2021a). There is robust evidence that adhesion-specific fibroblasts arise from MMT, from WT1 or Procr + mesothelium (Sandoval *et al.*, 2016; Fischer *et al.*, 2020; Zindel *et al.*, 2021a). Additional studies indicate that uninjured peritoneum may also contribute to adhesiogenesis, as opposing peritoneal surfaces can generate myofibroblasts and neovasculature in response to distant injury signals (Suzuki *et al.*, 2015), suggesting that adhesiogenesis involves coordinated responses across the peritoneal cavity rather than purely local fibroblast activation.

Thus, while submesothelial PDGFR^+^ fibroblasts are present and may contribute to fibrotic responses (particularly following chemical injury), current evidence suggests that MMT represents a predominant source of myofibroblasts after surgical injury. Whether similar mechanisms operate in endometriosis remains unclear.

### The role of fibrinogenesis and fibrinolysis in adhesion formation

Fibrin persistence results primarily from inhibition of anticoagulant pathways, and suppression of fibrinolysis through PAI-1 and repression of tPA/uPA activity. Here, the delicate balance between fibrinogenesis and fibrinolysis is disrupted, with the system tuned towards less fibrinolysis activity due to an abundance of inhibitors (Holmdahl, 1997; Hellebrekers and Kooistra, 2011; Rabieian *et al.*, 2018; Capella-Monsonís *et al.*, 2019). This is mediated via inflammation; peritoneal fibrinogenesis and fibrinolysis are modulated by many cytokines, including TGF-β, tumour necrosis factor-alpha (TNF-α), interleukin-1 (IL-1), and interleukin-6 (IL-6) (Holmdahl and Ivarsson, 1999; Holmdahl *et al.*, 2001; Cheong *et al.*, 2002b). TNF-α and IL-6 are pro-inflammatory cytokines involved in the acute-phase reaction, and studies demonstrate a positive correlation between peritoneal TNF-α and IL-6 concentrations and extent of pelvic adhesions (Cheong *et al.*, 2002b; Fredriksson *et al.*, 2014). Fibrin and fibrinogen stimulate TNF-α and IL-1β expression, which activates tissue factor. This prevents fibrinolysis by reducing tPA expression and increasing PAI-1 production by peritoneal mesothelial cells and endothelial cells (Capella-Monsonís *et al.*, 2019). Additionally, increased PAI-1 concentrations are associated with increased adhesion formation (Falk *et al.*, 2001; Di Filippo *et al.*, 2006; Honjo *et al.*, 2017), demonstrating the importance of plasminogen activation in adhesion prevention. This imbalance of fibrinogenesis and fibrinolysis is central to aberrant wound healing and adhesiogenesis and highlights the tight interplay between inflammation and coagulation (Fig. 4). These conserved mechanisms form the biological framework of adhesiogenesis across diverse forms of peritoneal injury. The following section examines how the endometriotic peritoneal environment may modify these pathways, thereby increasing susceptibility to adhesiogenesis in the absence of overt surgical trauma.

**Figure 4.**
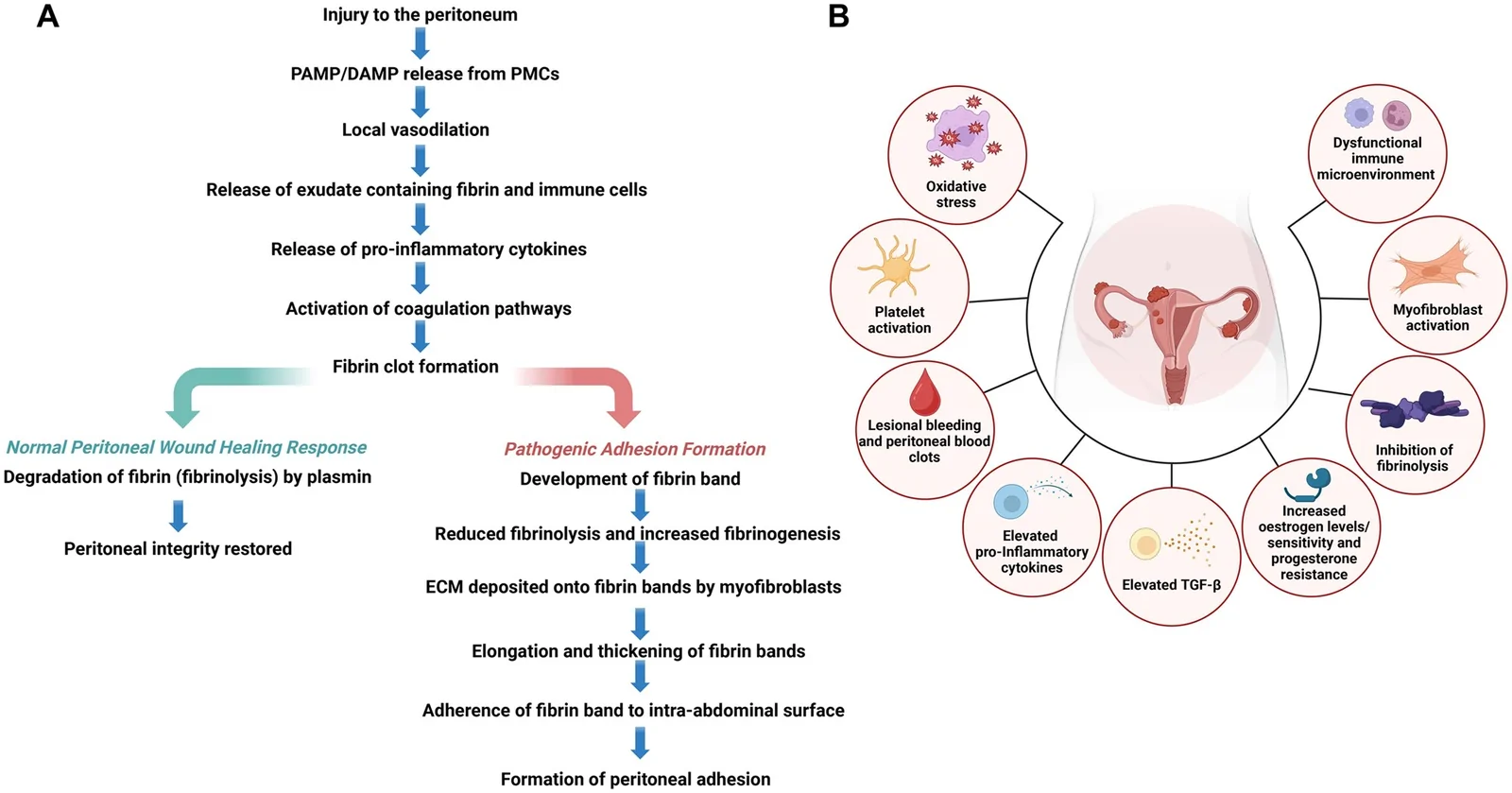
**Events of normal peritoneal wound-healing and pathogenic adhesion formation, and factors influencing endometriosis-related peritoneal adhesion development**. (**A**) A flow diagram demonstrating events of normal peritoneal wound-healing and pathogenic adhesion formation. Peritoneal damage results in PAMP and/or DAMP release from injured peritoneal mesothelial cells, in turn releasing vasoactive substances that promote vasodilation and release of a fibrous exudate. Activation of interdependent pro-inflammatory and coagulation pathways promotes fibrin clot formation and subsequent fibrin band development. In desired peritoneal wound-healing, fibrin bands are degraded. However, if the fibrinolytic capacity of the peritoneum is insufficient, fibrin bands become deposited in the extracellular matrix and fibrin, resulting in elongation of the fibrin band and adherence to an intra-peritoneal surface to form an adhesion (Mutsaers, 2002; Wang *et al*., 2008; Speyer and Ward, 2011; Zwicky *et al*., 2021). (**B**) An overview of the factors that may contribute to endometriosis-related adhesion formation. Abbreviations: DAMP, damage-associated molecular patterns; ECM, extracellular matrix; PAMP, pathogen-associated molecular patterns; PMC, peritoneal mesothelial cell; TGF-β1, transforming growth factor-beta 1. Created in BioRender. Greaves, E. (2026) https://BioRender.com/lkfxaw8.

## Peritoneal adhesion formation in endometriosis and related mechanisms

Endometriosis is commonly conceptualized as a condition of repeated tissue injury and repair within the peritoneal cavity, driven by cyclical endometriotic lesion activity, bleeding, and chronic inflammation. Unlike acute surgical injury, these recurrent insults may sustain thromboinflammatory signalling and prevent complete resolution of peritoneal wound-healing responses. Consequently, the peritoneal environment in endometriosis may become biased toward persistent fibrin deposition and fibrotic remodelling, thereby increasing susceptibility to adhesion formation.

Experimental models have been particularly informative in identifying candidate triggers of adhesion formation relevant to endometriosis. Early studies by Ryan and Majno demonstrated that peritoneal adhesion formation could be induced in rats by the presence of intraperitoneal blood clots (Ryan *et al.*, 1971; Jabbour *et al.*, 2006; Emera *et al.*, 2012), supporting the concept that fibrin-rich exudates can initiate adhesiogenesis. As some endometriotic lesions may exhibit cyclical bleeding (Laux-Biehlmann *et al.*, 2015), exposure of the peritoneal cavity to blood-derived components represents a plausible disease-specific stimulus for adhesiogenesis. Consistent with this, experimental exposure of murine peritoneum to increasing volumes of blood following establishment of CO_2_ pneumoperitoneum caused proportionally greater adhesion formation (Corona *et al.*, 2013). However, these models involve surgically introduced blood and concomitant peritoneal trauma, which may independently promote adhesiogenesis and therefore do not fully replicate the chronic, low-volume exposure to menstrual or endometriotic lesional blood occurring within the intact peritoneal cavity in endometriosis. Notably, no human studies have directly examined whether retrograde menstruation or endometriotic lesion-derived bleeding quantitatively influences adhesion formation. Determining whether repeated exposure to blood, iron, and fibrin degradation products within the closed peritoneal environment promotes pathological repair represents an important unmet research priority in endometriosis-associated adhesiogenesis.

The presence of endometrial tissue within the peritoneal cavity may itself represent a direct driver of adhesiogenesis. Experimental studies demonstrate that intraperitoneal injection of eutopic endometrial tissue from women with endometriosis into mice resulted in greater adhesion development compared with tissue derived from healthy controls (Stocks *et al.*, 2017), suggesting that disease-associated molecular alterations in endometrial tissue may promote adhesiogenesis. Supporting this concept, histological analysis of periovarian adhesions from endometriosis patients identified structures resembling endometrial tissue, including stromoglandular endometrioid cysts and tubules (Jirásek *et al.*, 1998), raising the possibility that ectopic endometrial cells may directly contribute to adhesion maturation. Together, these findings suggest refluxed or endometriotic lesion-derived endometrial tissue may function not only as a source of endometriotic lesion establishment but also as a local pro-fibrotic stimulus capable of extending fibrotic signalling beyond the endometriotic lesions and reinforcing pathological peritoneal repair in adjacent peritoneal tissue. However, it remains unclear whether endometriotic tissue initiates adhesiogenesis or preferentially incorporates into adhesions after they have formed, representing an important unresolved question in endometriosis-associated adhesiogenesis.

Establishment of endometriosis lesions induces multiple adaptations to the peritoneal microenvironment, including altered expression of steroid hormone signalling (Zeitoun *et al.*, 1998; Bulun *et al.*, 2000; Matsuzaki *et al.*, 2001; Zhang and Wang, 2023), oxidative stress (Santulli *et al.*, 2015; Zhang *et al.*, 2018), imbalance of pro-fibrotic and anti-fibrotic mediators (Pizzo *et al.*, 2002; Young *et al.*, 2014b), and altered peritoneal immunity (Braundmeier *et al.*, 2012; Henlon *et al.*, 2024) (Fig. 4). Rather than acting independently, these processes may collectively maintain a chronically activated peritoneal environment.

Endometriotic lesions comprise endometrial stromal cells, endometrial epithelial cells, vasculature, nerve fibres and immune cells (Zhang *et al.*, 2016a) and are characterized by progressive extracellular matrix deposition and fibrotic remodelling within the endometriotic lesion microenvironment (Garcia Garcia *et al.*, 2023). Endometriotic lesions reportedly undergo repeated cycles of injury and repair (Leyendecker and Wildt, 2011; Garcia Garcia *et al.*, 2023), promoting EMT, fibroblast-to-myofibroblast transition (FMT), smooth muscle metaplasia and fibrinogenesis (Ding *et al.*, 2015; Vissers *et al.*, 2024). Increased tissue stiffness and TGF-β1/Smad3 signalling further reinforce myofibroblast activation (Matsuzaki *et al.*, 2016; Zhang *et al.*, 2016b, 2019b; Vigano *et al.*, 2018), potentially generating a self-sustaining pro-fibrotic niche capable of influencing adjacent peritoneal repair processes. However, the role of myofibroblasts in endometriosis-related adhesiogenesis is unknown.

Direct investigation of endometriosis-associated adhesion formation in humans is limited by practical and ethical constraints; consequently, much mechanistic insight derives from experimental models, predominantly murine systems. These models typically require artificial induction of endometriosis through transplantation of endometrial tissue and hormonal manipulation, as spontaneous menstruation and endometriosis only occur in humans and certain primates (Emera *et al.*, 2012; Greaves *et al.*, 2014). Although murine models differ from human disease in immune composition and chronicity, they remain valuable for identifying biological mechanisms underlying adhesiogenesis. Accordingly, findings from experimental studies are interpreted cautiously and considered alongside available human data, with emphasis on mechanistic rather than direct clinical inference.

Although peritoneal adhesions are widely recognized as a feature of endometriosis, exact mechanisms driving their development in the absence of surgical trauma remain incompletely defined. Clinical observations demonstrating adhesions at the time of diagnosis suggest that endometriosis itself alters the conditions under which peritoneal repair occurs. Current evidence supports a model in which the endometriotic peritoneal environment sustains wound-healing processes that normally resolve after acute injury, thereby favouring fibrin persistence and fibroproliferative remodelling. In this context, recurrent endometriotic lesional activity, chronic inflammation, altered immune regulation, hormonal signalling, and impaired fibrinolysis may collectively shift peritoneal repair toward a state of incomplete resolution. Endometriotic lesions may therefore function as persistent sources of repair-associated signals, lowering the threshold for adhesion initiation and recurrence. Despite emerging associations between disease severity and adhesion burden (Barcz *et al.*, 2012; Yang *et al.*, 2025), the relationships between endometriotic lesion biology, adhesion development and clinical outcomes remain insufficiently defined. Clarifying these links will be essential to distinguish universal mechanisms of adhesiogenesis from those amplified in endometriosis and to guide future preventative strategies.

### Immune response in endometriosis-related adhesiogenesis

#### Role of peritoneal cytokines in endometriosis-related adhesiogenesis

Peritoneal injury triggers an acute inflammatory response characterized by rapid release of cytokines including TNF-α, IL-1, and IL-6 (Margetts *et al.*, 2002; Stadlmann *et al.*, 2006; Sammour *et al.*, 2010), which collectively initiate wound-healing pathways and promote expression of pro-adhesiogenic mediators such as TGF-β1, Col1a1, vascular endothelial growth factor (VEGF), and PAI-1 (Torres *et al.*, 2018; Uyama *et al.*, 2019; Hu *et al.*, 2021). Cytokine responses are largely compartmentalized within the peritoneal cavity, where concentrations exceed systemic levels and correlate with adhesion extent (Holmdahl *et al.*, 2001; Cheong *et al.*, 2002a,b; Hobson *et al.*, 2003; Sammour *et al.*, 2010; Fredriksson *et al.*, 2014). Notably, many of these same cytokines are chronically elevated in the peritoneal fluid of women with endometriosis, suggesting that disease-associated inflammation may sustain repair pathways normally restricted to acute injury (Lin *et al.*, 2018b).

Among these mediators, TGF-β1 is a central regulator of adhesiogenesis; promoting fibroblast proliferation, MMT, extracellular matrix deposition, and inhibition of fibrinolysis through increased PAI-1 and reduced tPA (Aroeira *et al.*, 2007; Sandoval *et al.*, 2016; Zhang *et al.*, 2016a; Rabieian *et al.*, 2018). Elevated TGF-β1 concentrations correlate with adhesion severity (Cheong *et al.*, 2002b), and experimental neutralization attenuates adhesion formation in animal models (Gorvy *et al.*, 2005). Furthermore, in women with a history of intra-abdominal surgery, elevated pre-operative serum TGF-β1 concentrations were predictive of intra-abdominal adhesions (Torres *et al.*, 2018). Temporal studies following surgery demonstrate biphasic TGF-β1 release, initially derived from platelet activation and subsequently from resident tissue cells, establishing a positive feedback loop that sustains myofibroblast activation and fibrotic remodelling (Wang *et al.*, 2014).

TGF-β1 is also a key driver of fibrosis in endometriotic lesions, where it promotes EMT and FMT, and extracellular matrix production (Zhu *et al.*, 2013; Vissers *et al.*, 2024). Elevated TGF-β1 concentrations are consistently reported in endometriotic tissue, peritoneal fluid, and serum of endometriosis patients relative to healthy controls (Table 1) (Pizzo *et al.*, 2002; Sikora *et al.*, 2018; Santoso *et al.*, 2021). Although its role in endometriotic lesion fibrosis is well established (Young *et al.*, 2014b), direct evidence linking TGF-β1 to adhesion formation in endometriosis remains limited; however, correlations between peritoneal TGF-β levels and adhesion components of the rASRM scores suggest shared fibrotic pathways (Santoso *et al.*, 2021).

**Table 1.** Concentrations of cytokines involved in adhesiogenesis in endometriosis patients.

Cytokine	EM	Control	*P-*value	Reference
**IL-6**	**Concentration EM (pg/ml) (n)**	**Control concentration (pg/ml) (n)**	***P-*value**	**Reference**
PF	20.7 (5.0–38.9)^a^ (n *=* 18)	5.0 (5.0–38.9)^a^ (n *=* 22)	0.015	Kalu *et al.* (2007)
	525.10 ± 1168.53^c^ (n *=* 33)	62.96 ± 82.35^c^ (n *=* 17)	0.0002	Drosdzol-Cop *et al.* (2012)
	69.28 ± 11.19^c^ (n *=* 201)	49.03 ± 6.19^c^ (n *=* 93)	<0.001	Li *et al.* (2021)
	32.3 (17.4–81.8)^b^ (n *=* 63)	15.7 (12.5–18.7)^b^ (n *=* 37)	<0.0001	Barcz *et al.* (2012)
Serum	45.82 ± 13.29^c^ (n *=* 31)	35.08 ± 4.39^c^ (n *=* 32)	<0.001	Li *et al.* (2021)
	21.58 (8.22, 60.36)^b^ (n *=* 20)	0.00 (0.00, 0.28)^b^ (n *=* 11)	0.001	Bedaiwy *et al.* (2002)
	*Mi*n*imal-mild EM:* 29.4 ± 9.0^c^ (n *=* 11)	17.8 ± 12.1^c^ (n *=* 72)	0.008	Martínez *et al.* (2007)
	*Moderate-severe EM:* 17.6 ± 10.3 (n *=* 36)			
	4.41 (1.47–15.01)^b^ (n *=* 68)	0.97 (0.29–2.98)^b^ (n *=* 70)	<0.001	Othman *et al.* (2008)
Plasma	0.71 (0–228.8)^a^ (n *=* 201)	0.34 (0–5.48)^a^ (n *=* 93)	<0.0001	Mihalyi *et al.* (2010)
	50.85^e^ (n *=* 19)	2.05^e^ (n *=* 25)	<0.001	Monsanto *et al.* (2016)
**IL-8**	**Concentration EM (pg/ml) (n)**	**Control Concentration (pg/ml) (n)**	***P-*value**	**Reference**
Plasma	1.77 (0–52.12)^a^ (n *=* 201)	0.875 (0–6.26)^a^ (n *=* 93)	<0.0001	Mihalyi *et al.* (2010)
	175.70^e^ (n *=* 19)	19.29^e^ (n *=* 25)	0.002	Monsanto *et al.* (2016)
PF	14.59 (9.57, 28.80)^b^ (n *=* 37)	1.04 (0.00, 9.02)^b^ (n *=* 25)	0.002	Bedaiwy *et al.* (2002)
	22.0 (16.0–27.2)^b^ (n *=* 50)	13.4 (10.9–19.9)^b^ (n *=* 35)	0.0002	Barcz *et al.* (2012)
**TNF-α**	**Concentration EM (pg/ml) (n)**	**Control Concentration (pg/ml) (n)**	***P-*value**	**Reference**
PF	5.2 (4.6–7.1)^a^ (n *=* 17)	8.0 (5.3–14.7)^a^ (n *=* 24)	0.053	Kalu *et al.* (2007)
	5.79 ± 5.60^c^ (n *=* 33)	1.68 ± 1.24^c^ (n *=* 17)	0.000001	Drosdzol-Cop *et al.* (2012)
	54.83 (26.25, 63.46)^b^ (n *=* 28)	0.00 (0.00, 0.00)^b^ (n = 12)	<0.001	Bedaiwy *et al.* (2002)
Plasma	0.03 (0–24.81)^a^ (n *=* 201)	0.44 (0–4.88)^a^ (n *=* 93)	<0.0001	Mihalyi *et al.* (2010)
	244.14^e^ (n *=* 19)	10.04^e^ (n *=* 25)	<0.001	Monsanto *et al.* (2016)
Serum	*EPP:* 2.02 (1.48–3.67)^b^ (n *=* 25)	*EPP:* 1.34 (1.20–1.79)^b^ (n *=* 13)	0.035	Xavier *et al.* (2006)
	*LPP:* 1.92 (1.49–3.07)^b^ (n *=* 25)	*LPP:*1.17 (1.03–1.99)^b^ (n *=* 13)	0.044	
	*ESP:* 1.89 (1.41–2.98)^b^ (n *=* 25)	*ESP:*1.31 (0.97–1.89)^b^ (n *=* 13)	0.035	
	*LSP:* 2.23 (1.29–3.64)^b^ (n *=* 25)	LSP: 1.28 (0.83–2.14)^b^ (n *=* 13)	0.024	
	8.33 (1.37, 69.22)^b^ (n *=* 21)	1.37 (1.37, 1.37)^b^ (n *=* 22)	0.003	Bedaiwy *et al.* (2002)
**TGF-β1**	**Concentration EM (ng/ml) (n)**	**Control Concentration (ng/ml) (n)**	***P-*value**	**Reference**
Serum	37.4 ± 2.7^d^ (n *=* 120)	21.6 ± 2.7^d^ (n *=* 89)	<0.0001	Lee *et al.* (2011)
PF	1.12 ± 0.57^c^ (n *=* 12)	0.85 ± 0.26^c^ (n *=* 10)	<0.05	Hanada *et al.* (2018)
	61.19 ± 8.81^d^ (n *=* 30)	24.88 ± 6.98^d^ (n *=* 32)	<0.001	Guo *et al.* (2016)
	164 (15–289)^a^ (n *=* 33)	15 (15–235)^a^ (n *=* 10)	<0.0001	Santoso *et al.* (2021)
**VEGF**	**Concentration EM (pg/ml) (n)**	**Control concentration (pg/ml) (n)**	***P-*value**	**Reference**
Serum	94.28 ± 13.02^c^ (n *=* 31)	78.21 ± 13.29^c^ (n *=* 32)	<0.001	Li *et al.* (2021)
	*ESP:* 238 (160–396)^b^ (n *=* 25)	*ESP:* 189 (110–221)^b^ (n *=* 13)	0.034	Xavier *et al.* (2006)
	*LSP:* 261 (173–404)^b^ (n *=* 25)	*LSP:* 199 (99–246)^b^ (n *=* 13)	0.019	
	10.15 ± 2.07^c^ pg/l (n *=* 80)	6.94 ± 1.25^c^ pg/l (n *=* 40)	<0.05	Zhang *et al.* (2018)
Plasma	3.12 (0.18–12.7)^a^ (n *=* 168)	0.27 (0.18–6.4)^a^ (n *=* 92)	0.0128	Vodolazkaia *et al.* (2016)
PF	110.23 ± 23.29^c^ (n *=* 31)	92.01 ± 14.39^c^ (n *=* 32)	<0.001	Li *et al.* (2021)

Pooled data are displayed from relevant studies comparing cytokine concentrations between endometriosis patients and controls, in serum, plasma, and peritoneal fluid. Abbreviations: EM, endometriosis; EPP, early proliferative phase; ESP, early secretory phase; IL-6, interleukin-6; IL-8, interleukin-8; LPP, late proliferative phase; LSP, late secretory phase; ng/ml, nanogram per millilitre; PF, peritoneal fluid; pg/ml, picogram per millilitre; pg/l, picogram per litre; TNF-α, tumour necrosis factor alpha; TGF-β1, transforming growth factor-beta 1; VEGF, vascular endothelial growth factor. *a: Media*n (*range*)*, b: Median* (25th *percentile*, 75th *percentile*)*, c: Mean ± SD, d: Mean ± SEM, e: Mean*.

Additional inflammatory cytokines further reinforce this pro-adhesiogenic environment. IL-1, IL-6, and interleukin-8 are elevated in adhesion tissue and peritoneal fluid of women with endometriosis (Table 1), where they impair fibrinolysis through PAI-1 activation (Cheong *et al.*, 2002b; Barcz *et al.*, 2012; Nishimoto-Kakiuchi *et al.*, 2023). Experimental inhibition of inflammatory signalling reduces fibrosis and adhesion formation in animal models, supporting a functional role for persistent cytokine activation. Similarly, TNF-α promotes inflammatory signalling, cellular adhesion, and EMT, and blockade of TNF signalling prevents adhesion development in primate models of endometriosis (D’Hooghe *et al.*, 2006). Collectively, these findings support a model in which endometriosis-associated inflammation sustains cytokine signalling networks that normally resolve after injury, thereby maintaining a pro-fibrotic, hypofibrinolytic peritoneal environment conducive to adhesiogenesis.

#### The role of macrophages in endometriosis-related adhesiogenesis

Macrophages are central regulators of peritoneal repair, and their functional state may determine whether healing resolves normally or progresses toward adhesiogenesis. During peritoneal injury, macrophages exhibit substantial functional plasticity, transitioning between pro-inflammatory and pro-repair states that coordinate inflammation, fibrinolysis, and tissue remodelling (Hong *et al.*, 2015; Sandoval *et al.*, 2016). Pro-inflammatory macrophages promote adhesiogenesis through secretion of cytokines such as TNF-α and IL-6 and by enhancing mesothelial and fibroblast activation. Pro-repair macrophages may limit adhesiogenesis by promoting fibrinolysis and matrix remodelling, partly through release of hepatocyte growth factor, and subsequent downregulation of PAI-1 (Ohashi *et al.*, 2014; Iwasaki *et al.*, 2019). Experimental studies demonstrate that promoting pro-repair macrophage polarization attenuates adhesion formation, supporting a role for macrophages as a key determinant of repair outcome (Hong *et al.*, 2015). In the context of endometriosis, where dynamic endometriotic lesion activity and inflammation may repeatedly activate peritoneal repair pathways, macrophage-driven resolution mechanisms may be persistently engaged yet incompletely resolved, potentially favouring fibroproliferative remodelling over full tissue restitution.

Despite this protective role, macrophages are also detected in mature human adhesions, suggesting functions extending beyond initiation to long-term tissue remodelling (Binnebösel *et al.*, 2008). Notably, human studies and experimental endometriosis models demonstrate that macrophages within the peritoneal cavity are predominantly skewed toward pro-repair phenotypes, highlighting a paradox in which reparative signalling coexists with progressive fibrosis. This suggests that persistent or dysregulated repair responses, rather than sustained classical inflammation alone, may predispose the endometriotic peritoneum to adhesiogenesis. Consistent with this, scar-associated macrophages (SAMs) are seen in both endometriosis lesions and peritoneal adhesions in experimental mouse models (Zindel *et al.*, 2021a; Henlon *et al.*, 2024), suggesting that shared macrophage activation states may link endometriotic lesion-associated repair processes with adhesion formation through common pro-fibrotic mechanisms.

In murine injury models, evidence indicates that macrophage effects on adhesion formation are highly context-dependent. Peritoneal injury induces rapid inflammatory monocyte recruitment, alongside a transient reduction in resident macrophages (the MDR), and is followed by niche replenishment (Rojo and Conget, 2018; Tsai *et al.*, 2019). Some studies report that inhibition of macrophage recruitment reduces peritoneal adhesion development (Hoshino *et al.*, 2007; Tsai *et al.*, 2019; Ito *et al.*, 2021), whereas others report that macrophage depletion or inhibition of monocyte recruitment increases adhesion formation (Burnett *et al.*, 2006; Sahputra *et al.*, 2022). These seemingly conflicting findings likely reflect differences in injury models (surgical, ischaemic button, induced colitis), timing of depletion, and macrophage subsets targeted, suggesting that macrophages may exert both protective and pro-fibrotic roles depending on the phase of repair. Importantly, most of these studies model acute peritoneal trauma rather than chronic disease environments like endometriosis.

In endometriosis, macrophage biology is further altered by sustained monocyte recruitment and endometriotic lesion-derived signalling. Macrophage numbers are elevated in the peritoneal cavity of endometriosis patients and mice with experimental endometriosis, and are maintained by continuous monocyte trafficking (Bersinger *et al.*, 2009; Guo *et al.*, 2020; Hogg *et al.*, 2020). Single-cell profiling identified multiple macrophage populations within endometriotic lesions, including monocyte-derived populations that differentiate *in situ* to give rise to prodisease macrophage populations resembling SAMs and tumour-associated macrophages (TAMs) (Henlon *et al.*, 2024). These macrophages secrete cytokines and growth factors, including TGF-β and VEGF (Symons *et al.*, 2018), which support endometriotic lesion persistence and may simultaneously reinforce pro-fibrotic repair pathways. Thus, the endometriotic niche may reshape macrophage function toward sustained tissue remodelling, lowering the threshold for adhesiogenesis.

Beyond cytokine signalling, macrophages may directly regulate early adhesion initiation through physical interactions with injured peritoneum. In murine models, resident peritoneal macrophages rapidly aggregate at sites of mesothelial injury, forming a transient cellular barrier that covers fibrin deposits until remesothelialization occurs (Ito *et al.*, 2021). Incomplete coverage of fibrin clots is associated with peritoneal adhesion formation, whereas enhancement of macrophage accumulation improves fibrin shielding and attenuates adhesiogenesis (Ito *et al.*, 2021). The protective effect of macrophage accumulation at injured mesothelium is consistent with intravital imaging results that demonstrate rapid aggregation of GATA6^+^ resident macrophages following thermal peritoneal injury, in a manner synonymous with platelet aggregation during thrombus formation. Excessive aggregation following severe injury or more complex insults can generate ‘super-aggregates’ associated with collagen deposition and adhesiogenesis. Consistently, inhibition of GATA6^+^ peritoneal resident macrophage aggregation reduced adhesion formation in mice (Zindel *et al.*, 2021b). Whether similar mechanisms operate during endometriotic lesion establishment remains unknown, but persistent inflammatory stimuli may favour maladaptive macrophage aggregation and prolonged fibrin exposure. Together, current evidence suggests macrophages act as critical regulators of peritoneal repair balance; chronic inflammatory signalling and continuous immune recruitment may shift macrophage function from transient repair coordination toward sustained fibroproliferative activity, thereby promoting adhesion susceptibility. Defining human peritoneal macrophage subsets and their functional roles during endometriosis-associated repair represents an important priority for understanding disease-specific mechanisms of adhesiogenesis.

### The effect of peritoneal hypoxia on endometriosis-related adhesiogenesis

Hypoxia represents an important microenvironmental regulator of peritoneal repair. While transient hypoxia occurs following acute surgical injury, endometriosis may generate sustained hypoxic signalling through cyclical inflammation, endometriotic lesion-associated bleeding and accumulation of iron derived from retrograde menstruation (Szczepańska *et al.*, 2003), and macrophage haem metabolism (Defrère *et al.*, 2008). These processes increase ROS production within the peritoneal cavity, and elevated oxidative stress markers have consistently been identified in the peritoneal fluid of women with endometriosis compared with controls (Santulli *et al.*, 2015).

Cellular responses to hypoxia are primarily mediated through hypoxia-inducible factor 1-alpha (HIF-1α), which regulates inflammatory signalling, angiogenesis, and cellular survival pathways. Experimental studies demonstrate that hypoxia promotes expression of pro-adhesiogenic mediators including TGF-β, TNF-α, IL-6, Col1a1, and VEGF, while simultaneously disrupting the balance between fibrinogenesis and fibrinolysis (Saed and Diamond, 2004; Ambler *et al.*, 2012a; Fletcher *et al.*, 2015; Capella-Monsonís *et al.*, 2019). Under hypoxic conditions, human peritoneal fibroblasts exhibit reduced tPA expression and increased PAI activity (Saed and Diamond, 2003), indicating impaired fibrinolytic capacity and enhanced fibrotic potential. In line with this, HIF-1α expression correlates with adhesion severity in human tissues (Biller *et al.*, 2022).

Hypoxia is also a recognized component of endometriosis pathobiology. Increased HIF-1α and VEGF expression have been identified in endometriotic lesions and the circulation of endometriosis patients, correlating with rASRM stage (Young *et al.*, 2014a; Zhang *et al.*, 2018). In endometriotic cells, hypoxia promotes EMT and autophagy-dependent survival pathways, processes associated with myofibroblast generation and extracellular matrix deposition (Liu *et al.*, 2018). Although direct evidence linking hypoxia to adhesiogenesis in humans remains limited, experimental reduction of oxidative stress decreases adhesion formation in animal models, supporting a contributory role for ROS-driven repair dysregulation. Together, these findings suggest sustained hypoxia and oxidative stress in endometriosis may prevent normal resolution of peritoneal wound healing, reinforcing a persistent repair phenotype characterized by impaired fibrinolysis and enhanced fibroproliferative remodelling.

### Coagulation and fibrinolytic imbalance in endometriosis-associated adhesiogenesis

Resolution of peritoneal injury depends on timely degradation of fibrin deposits. A central feature of adhesion formation is therefore hypofibrinolysis, permitting persistence of fibrin scaffolds that support extracellular matrix deposition. Several studies suggest that endometriosis is associated with systemic and local alterations in haemostatic regulation, including increased platelet activation, elevated levels of fibrinogen and tissue factor, and reduced thrombin time, consistent with a hypercoagulable phenotype (Ding *et al.*, 2018; Lin *et al.*, 2018a; Viganò *et al.*, 2018).

Particular attention has focused on PAI-1, a key regulator of fibrinolysis. Increased PAI-1 expression has been reported in endometriotic tissue and peritoneal fluid, with higher concentrations associated with the presence of adhesions in affected individuals (Bruse *et al.*, 1998, 2005; Hellebrekers *et al.*, 2005; Gilabert-Estellés *et al.*, 2007). Genetic polymorphisms linked to increased PAI-1 transcription further support a hypofibrinolytic environment in subsets of women with endometriosis (Bedaiwy *et al.*, 2006). Persistence of fibrin deposits under these conditions may facilitate both endometriotic lesion implantation and adhesion development by stabilizing provisional extracellular matrices. However, there is disputing evidence surrounding PAI-1 polymorphisms and susceptibility to endometriosis (Gentilini *et al.*, 2009; Zhao *et al.*, 2016; Li *et al.*, 2023), and genome-wide association study-level data is required given the risk of false positives in single-nucleotide polymorphism associations within independent cohorts.

Conversely, increased uPA expression observed in endometriotic tissue may promote invasion and endometriotic lesion establishment, highlighting a context-dependent imbalance between fibrin stability and matrix degradation (Fernández-Shaw *et al.*, 1995; Bruse *et al.*, 1998, 2004; Yoshida *et al.*, 2004; Ramón *et al.*, 2005; Buigues *et al.*, 2018). While these alterations may favour endometriotic lesion survival, their direct contribution to adhesion formation remains incompletely defined, but available evidence suggests endometriosis shifts the coagulation–fibrinolytic balance toward fibrin persistence, increasing propensity for adhesiogenesis when peritoneal repair is repeatedly activated.

### Hormonal regulation of adhesiogenesis: the role of oestrogen

Ectopic endometrial tissue exhibits enhanced local oestradiol production through aberrant expression of steroidogenic enzymes, together with progesterone resistance that limits normal anti-proliferative signalling (Zeitoun *et al.*, 1998; Osiński *et al.*, 2018; Hogg *et al.*, 2020). These alterations create a persistently oestrogen-dominant microenvironment within the peritoneal cavity.

Oestradiol regulates multiple pathways implicated in fibrosis and adhesiogenesis, including Wingless/Beta-catenin (Wnt/β-catenin), TGF-β1, Rho-kinase, and Notch signalling (Kanzaki *et al.*, 1995; Ono *et al.*, 2015; Han *et al.*, 2019; Zhu *et al.*, 2023). Activation of these pathways promotes EMT, fibroblast activation, and extracellular matrix production in endometriotic cells (Ono *et al.*, 2015; Zhu *et al.*, 2023). Notably, Wnt/β-catenin signalling is increased both in endometriotic fibrosis and adhesion tissues, suggesting shared downstream mechanisms linking endometriotic lesion biology with adhesion development (Binnebösel *et al.*, 2008; Foster *et al.*, 2020).

Experimental evidence further supports hormonal influence on adhesiogenesis: suppression of oestrogen production reduces adhesion formation in primate models (Poirier *et al.*, 2022) and sex steroid receptors are expressed in human pelvic adhesion tissue, indicating direct hormonal responsiveness (Wiczyk *et al.*, 1998). Elevated oestradiol may therefore enhance sensitivity of peritoneal fibroblasts to pro-fibrotic signalling, amplifying repair responses triggered by injury or inflammation. Taken together, oestrogen signalling appears to act as an upstream regulator that sustains pro-fibrotic pathways in endometriosis, reinforcing conditions that favour persistent repair activation and adhesiogenesis.

Collectively, the mechanisms described above suggest that peritoneal adhesions in endometriosis arise not from fundamentally distinct biological pathways, but from dysregulated activation of normal peritoneal repair processes. Dynamic endometriotic lesion activity, inflammation and bleeding repeatedly engage wound-healing responses that would ordinarily resolve following acute injury. Within this altered microenvironment, sustained hypoxic and oxidative stress signalling impairs resolution of repair, haemostatic imbalance promotes persistence of fibrin scaffolds, and oestrogen-dependent signalling amplifies pro-fibrotic cellular responses. Together, these interacting factors shift peritoneal healing toward a state of incomplete resolution characterized by persistent extracellular matrix deposition and fibroproliferative remodelling. In this context, endometriosis may be conceptualized as a condition in which repeated activation of physiological repair pathways lowers the threshold for adhesiogenesis, thereby linking endometriotic lesion biology with the development and recurrence of peritoneal adhesions.

## Clinical implications of peritoneal adhesions in endometriosis

Endometriosis-associated peritoneal adhesions may arise following surgery and also from the inflammatory and fibrotic endometriosis disease process itself. Both types are associated with substantial morbidity and increased healthcare utilization (Abd El-Kader *et al.*, 2019). In women with severe endometriosis, pelvic adhesions are reported in 50–100% of cases (Parker *et al.*, 2005; Dehbashi *et al.*, 2019). Consistent with high clinical burden, gynaecological surgeons identify endometriosis surgery (alongside myomectomy) as among the procedures most commonly complicated by adhesion formation (Wallwiener *et al.*, 2014) (Fig. 5). Although laparoscopic surgery reduces risk of adhesion-related readmissions, the overall burden of adhesion-associated morbidity remains substantial, with approximately 27% of patients readmitted within 5 years for adhesion-related causes or reoperations (Krielen *et al.*, 2020). Currently, meta-analyses concerning adhesion prevalence in endometriosis are lacking. However, pooled data from relevant studies is compiled in Table 2.

**Figure 5.**
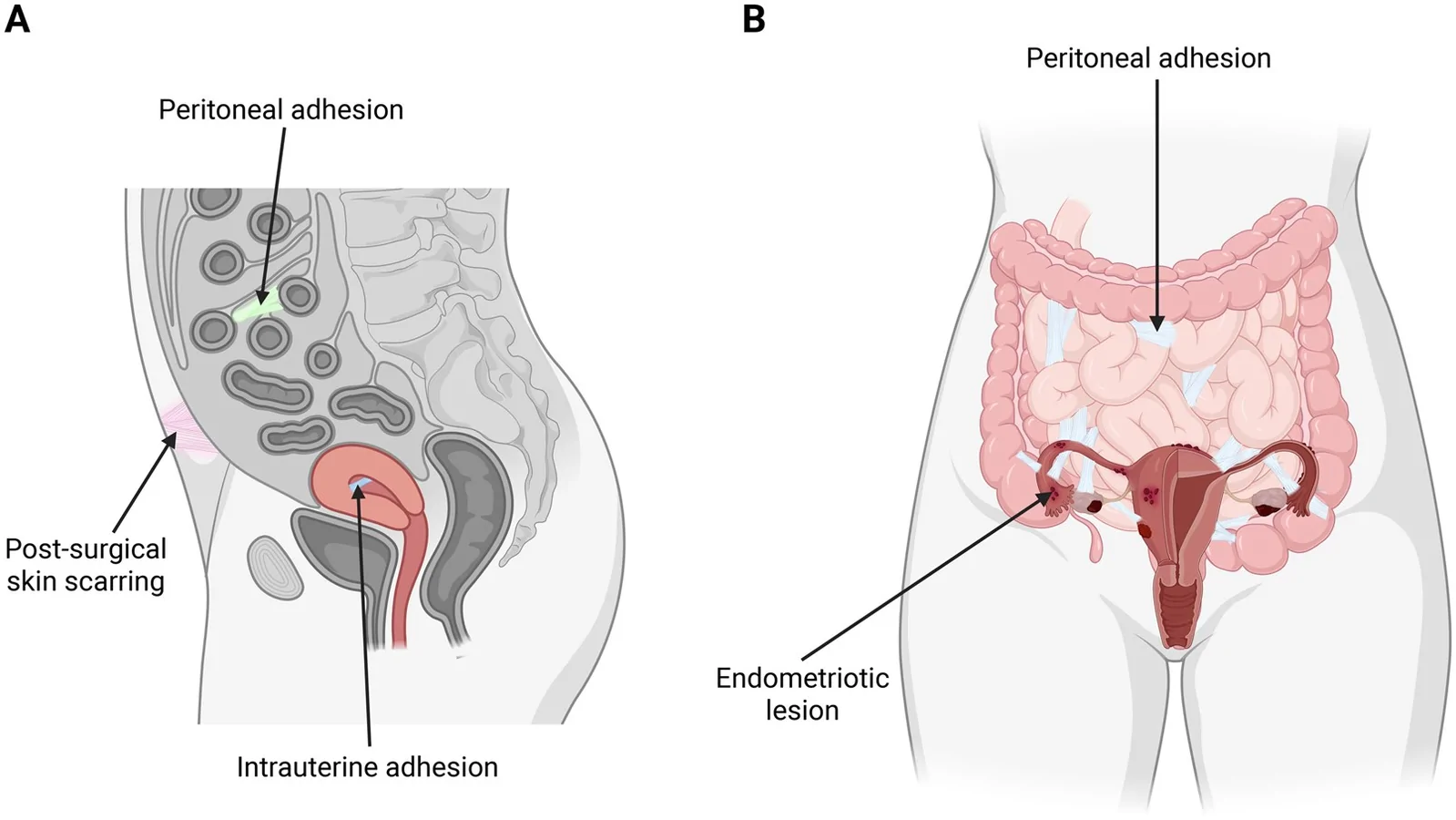
**Types of adhesions in endometriosis and other gynaecological conditions**. (**A**) An illustration demonstrating the location of peritoneal adhesions, within the peritoneal cavity, and intrauterine adhesions, within the uterus, of the female pelvic cavity. Post-surgical skin scarring, distinct from adhesion formation, is present following caesarean section or alternative abdominopelvic surgeries. Figure adapted from Zindel (2024) using BioRender.com. (**B**) The abdominopelvic cavity of a patient with endometriosis, demonstrating endometriotic lesions and peritoneal adhesions. Created in BioRender. Greaves, E. (2026) https://BioRender.com/bldot2p.

**Table 2.** The prevalence of adhesions in endometriosis patients.

	*%*	n	Reference
Adhesions detected by laparoscopy			
No previous endometriosis surgery	75.0	6	Parker *et al.* (2005)
	33.0	93	Holdsworth-Carson *et al.* (2024)
	37.6	41	Abd El-Kader *et al.* (2019)
	61.5	40	Barcz *et al.* (2012)
	81.9	470	Parazzini *et al.* (2006)
Previous endometriosis surgery	82.0	31	Parker *et al.* (2005)
	59.3	131	Holdsworth-Carson *et al.* (2024)
Unknown surgical history	39.4	78	Holland *et al.* (2013)
	74.0	20	Koninckx *et al.* (2016)
	92.0	161	Setälä *et al.* (2011)
	100	10	Korell *et al.* (2016)
Adhesions detected by TVUSS after laparoscopic endometriosis surgery			
	86.0	38	Seracchioli *et al.* (2014)
	51.9	27	Hoo *et al.* (2014)
	68.8 [ovarian adhesions]	11	Hoo *et al.* (2011)
	70.0 [adnexal adhesions]	28	Fraser *et al.* (2015)
Adhesions detected by site	*%*	n	Reference
Adnexal	51.2	21	Abd El-Kader *et al.* (2019)
	70.0	28	Fraser *et al.* (2015)
Anterior abdominal wall	24.4	10	Abd El-Kader *et al.* (2019)
Vesico-uterine	12.2	5	Abd El-Kader *et al.* (2019)
	1.81	1	Tanaka *et al.* (2025)
Uterus to abdominal wall	9.80	4	Abd El-Kader *et al.* (2019)
Uterus and left ovary	67.3	37	Tanaka *et al.* (2025)
Uterus and right ovary	56.4	31	Tanaka *et al.* (2025)
Uterus and rectum	60.0	33	Tanaka *et al.* (2025)
Right ovary and left ovary	10.9	6	Tanaka *et al.* (2025)
Left ovary and rectum	32.7	18	Tanaka *et al.* (2025)
Right ovary and rectum	23.6	13	Tanaka *et al.* (2025)
Tubal	80.0	64	Rani *et al.* (2022)
Ovarian	18.8	15	Rani *et al.* (2022)
	68.8	11	Hoo *et al.* (2011)
Adhesions by rASRM stage			
Stage I	50.4	66 (*P* = 0.17)	Yang *et al.* (2025)
Stage II	57.1	32 (*P* = 0.17)	Yang *et al.* (2025)
Stage III	63.0	29 (*P* = 0.17)	Yang *et al.* (2025)
Stage IV	68.2	28 (*P* = 0.17)	Yang *et al.* (2025)
Stages I–II	44.0	11 (*P* = 0.037)	Barcz *et al.* (2012)
Stages III–IV	72.5	29 (*P* = 0.037)	Barcz *et al.* (2012)

This table provides pooled data from relevant studies on the prevalence of adhesions detected by laparoscopy, or TVUSS, the prevalence of adhesions at various sites, and the prevalence of adhesions in different categories of the rASRM staging system. Abbreviations: rASRM, revised American Society for Reproductive Medicine; TVUSS, transvaginal ultrasound scan.

Adhesions can distort abdominopelvic anatomy, contributing to small bowel obstruction, chronic abdominopelvic pain, and reproductive dysfunction. Importantly, unlike many proposed mechanisms linking endometriosis to infertility, the adverse effects of adhesions on spontaneous conception are biologically intuitive and largely mechanical. During natural conception, adhesions can impair tubo-ovarian mobility, compromise fimbrial oocyte pick-up, and disrupt gamete transport, thereby contributing to subfertility, infertility, and ectopic pregnancy (Tanbo and Fedorcsak, 2017; Lin *et al.*, 2018b). Specifically, peritubal and periovarian adhesions may prevent normal apposition of fimbriae to the ovary, restrict ovulatory follicle rupture and ovum release, and impair transport of gametes and embryos through the reproductive tract. Consequently, fertility may be compromised even in the presence of patent fallopian tubes, highlighting that anatomical distortion can produce a functional impairment of reproductive capacity that is not readily detected using conventional tubal patency testing (Tulandi *et al.*, 1990). Furthermore, in assisted reproduction, dense adhesions may complicate oocyte retrieval because of reduced ovarian accessibility, altered ovarian position and proximity of adjacent organs, potentially increasing procedural complexity and risk. However, because *in vitro* fertilization largely bypasses the requirement for fimbrial oocyte capture and tubal transport, the reproductive consequences of adhesions are likely to be more profound for natural conception than for assisted conception (Practice Committee of the American Society for Reproductive Medicine, 2012).

There is limited data on the impact of adhesions on fertility in endometriosis patients, outlining an area for future research. Evidence supporting a direct causal relationship between adhesions and infertility remains limited, partly because adhesion severity frequently correlates with overall disease burden. Nevertheless, observational studies have reported improved spontaneous pregnancy rates following laparoscopic adhesiolysis, suggesting restoration of normal pelvic anatomy may improve fecundity in selected patients (Gomel, 1983; Tulandi *et al.*, 1990). However, the quality of evidence remains low and contemporary prospective studies in endometriosis populations are lacking. Taken together, adhesions represent one of the most plausible mechanisms through which endometriosis may impair fertility by physically disrupting reproductive anatomy; however, their independent contribution to infertility remains poorly quantified because adhesion severity frequently correlates with overall disease burden, making it difficult to disentangle the effects of adhesions from those of concomitant lesions, inflammation, and altered peritoneal physiology.

Pelvic adhesions may exacerbate endometriosis-associated pain through both mechanical and neurobiological mechanisms (Abd El-Kader *et al.*, 2019; Cofini *et al.*, 2024). In keeping with this, moderate-to-severe adhesions have been associated with a 4.2-fold increased risk of severe dysmenorrhoea relative to endometriosis patients without adhesions (Forman *et al.*, 1993). Furthermore, adhesion-related symptoms can be difficult to validate without surgery, which may compound patient distress and clinical uncertainty (Tabibian *et al.*, 2017).

Adhesions also increase operative complexity during endometriosis surgery (Abd El-Kader *et al.*, 2019). Patients undergoing adhesiolysis have higher rates of intraoperative complications (Clark *et al.*, 2020), likely due to impaired visibility, distorted anatomy, and risk of inadvertent organ injury. Conversion to laparotomy may be required in difficult cases, which itself increases risk of further adhesion formation (Tabibian *et al.*, 2017). Despite the frequency of adhesiolysis in endometriosis surgery, high-quality data evaluating long-term outcomes and adhesion reformation rates remain limited.

Mechanistically, pain-transmitting nerve fibres have been identified within human adhesions, and adhesion palpation during awake laparoscopy can reproduce pain (Sulaiman *et al.*, 2001; Demco, 2004). In ovarian endometriosis, pelvic adhesions have been associated with higher densities of sensory nerve fibres (Zhang *et al.*, 2010), which positively correlated with pain severity (Yao *et al.*, 2010). Finally, adhesions present at diagnosis may stratify recurrence risk: 5 years after laparoscopic diagnosis of endometriosis, women without adhesions had a higher probability of remaining recurrence-free than those with adhesions (Holdsworth-Carson *et al.*, 2024), suggesting that adhesions may represent both a marker and a potential facilitator of disease chronicity. Together, these observations emphasize adhesions as a clinically meaningful component of the endometriosis phenotype; however, the mechanisms driving adhesiogenesis in the absence of surgical trauma remain incompletely defined. Dissecting which steps of adhesiogenesis reflect conserved peritoneal wound-healing biology versus endometriosis-specific modifiers is therefore essential.

## Therapeutic strategies for endometriosis-related adhesions

### Adhesiolysis

The management of adhesions in endometriosis is challenging, and effective pharmacotherapies remain unavailable, rendering surgery the mainstay of treatment (Tabibian *et al.*, 2017). Adhesiolysis may restore anatomical relationships, facilitate access to deep infiltrating disease, relieve mechanical bowel or adnexal fixation, and potentially improve fertility in selected patients. In symptomatic deep infiltrating disease, surgical treatment is often required due to progressive dense adhesions, which can cause organ dysfunction and ‘frozen pelvis’ (Abd El-Kader *et al.*, 2019). However, its benefit for chronic pelvic pain remains inconsistent, and patient selection is critical. Elective adhesiolysis was ineffective in treating chronic pain attributed to adhesions when no barrier agent (agents used to mechanically separate peritoneal structures to permit peritoneal healing) was used (Swank *et al.*, 2003). Importantly, adhesiolysis is associated with a high rate of adhesion reformation, reported to be up to 97% in some series where no barrier agent was used (Diamond, 1991, 2000). Furthermore, repeat surgery increases operative complexity, risk of organ injury, and further adhesion formation.

### Surgical adaptations for prevention of adhesions

Several methods employed during endometriosis surgery reduce risk of post-operative adhesion formation (Somigliana *et al.*, 2012). Meticulous surgical technique remains the most important and universally applicable preventive strategy. Increased blood loss during surgery was associated with increased adhesion incidence (Trew *et al.*, 2011), and thus limiting surgical trauma is crucial. Other practices include adequate irrigation, minimal cautery to limit trauma, optimum suture material, and application of talcum-free and latex-free gloves (Diamond *et al.*, 2010; Arung *et al.*, 2011). In a cohort of 414 European gynaecological surgeons, only 44.3% of respondents regularly used anti-adhesion agents, and only 60% purported knowledge of surgical methods to reduce adhesion formation, indicating a need for education on adhesion prevention (Wallwiener *et al.*, 2014).

Minimally invasive surgery (MIS) reduces adhesion formation compared with laparotomy, largely due to reduced tissue trauma and smaller incisions (Lundorff *et al.*, 1991). For this reason, MIS is the preferred surgical approach in endometriosis whenever technically feasible. However, MIS is not adhesion-neutral. Pneumoperitoneum with cold, dry CO_2_ causes rapid peritoneal cooling and alters intra-abdominal pressure, causing bulging of peritoneal mesothelial cells, basement membrane exposure, and immune cell migration (Liu and Hou, 2006). Decreased temperatures increase peritoneal PAI-1 concentrations (Brokelman *et al.*, 2008); Therefore, basement membrane exposure and inflammatory and fibrinolytic pathway alterations may increase the risk of adhesion formation during laparoscopy. Moreover, duration of pneumoperitoneum positively correlated with number of post-operative adhesions (Brokelman *et al.*, 2011).

### Use of barrier agents for endometriosis-related adhesion prevention

Mechanical barriers promote normal peritoneal healing by temporarily separating peritoneal structures for the duration of normal peritoneal healing time (3–5 days), before resorption to prevent local and systemic inflammatory reactions (Koninckx *et al.*, 2016; Krämer *et al.*, 2021) (Table 3). The most widely used barrier agents include: Seprafilm (sodium hyaluronate/carboxymethylcellulose) and Interceed (oxidized regenerated cellulose) (Krämer *et al.*, 2021). Evidence from general gynaecological surgery supports their efficacy in reducing adhesion formation, and studies in visceral surgery patients indicate that barriers are effective in reducing risk for readmission due to acute small bowel obstruction (van den Beukel *et al.*, 2023). However, evidence specific to endometriosis surgery is more limited and heterogeneous. Key limitations of barrier agents include the challenge of predicting sites of potential adhesion formation (De Wilde *et al.*, 2016), the challenges of laparoscopic application, and reduced efficacy in the presence of bleeding.

**Table 3.** Summary of barrier agents used for adhesion prevention in gynaecological surgery.

Barrier agent	Advantages	Limitations	References
Oxiplex/AP Gel (polyethylene oxide and carboxymethylcellulose)	Effect haemostatic agent	Long-resorption time of up to 6 weeks	Young *et al.* (2005)
		Residual gelatinous collections reported in stage IV endometriosis	Young *et al.* (2005)
Hyaluronic acid (an extracellular matrix protein)	Ease of application enables wide coverage	Rapid clearance from peritoneal cavity	Yeo *et al.* (2006)
	Downregulates inflammation, stimulates fibrinolysis and promotes PMC proliferation		Yeo *et al.* (2006) Adamyan *et al.* (2024)
New cross-linked hyaluronan gel	Anti-proliferative activity at endometriotic lesion sites		Ekin *et al.* (2021)
	Reduced endometriosis-related symptoms 6 months post-operatively		
Seprafilm (composed of sodium hyaluronate and carboxymethylcellulose)	Resorption time of 7 days	Laparoscopic application is challenging	Pados *et al.* (2010) Krämer *et al.* (2021)
		Poor coverage on uneven surfaces	Kiefer *et al.* (2016)
		Use in laparoscopic surgery is challenging due to fragility	Diamond *et al.* (2010)
4DryField^®^PH (manufactured from purified potato starch)	Haemostat activity when applied as a powder	Irrigation with saline required to transform gel	Krämer *et al.* (2021) Ziegler and De Wilde (2022)
	Resorption time of 7 days post-operatively	Excessive application without irrigation can cause debris granuloma formation and peritoneal inflammation	Krämer *et al.* (2021)
Interceed (composed of oxidized regenerated cellulose)	Effective at separating tissues	May increase PA formation	Fazel Anvari-Yazdi *et al.* (2025) Schaefer *et al.* (2024)
	Biodegradable	Presence of blood impairs anti-adhesion effects	
		Able to capture blood from injured sites, causing accumulation of fibrin deposits within mesh	Krämer *et al.* (2021)
		Long resorption time of 4 weeks	

This table summarizes the reported advantages and disadvantages of various barrier agents used to prevent adhesion formation in gynaecological surgery. Abbreviations: PA, peritoneal adhesion; PMC, peritoneal mesothelial cell.

Oxiplex/AP (polyethylene oxide and carboxymethylcellulose) decreased post-operative adhesion formation by 32% in laparoscopic gynaecological surgery (Young *et al.*, 2005). In endometriosis surgery, Oxiplex/AP gel reduced adhesion formation relative to controls. However, not all participants had histological confirmation of endometriosis (diZerega *et al.*, 2007). A novel barrier agent, 4DryField^®^PH is manufactured from purified potato starch and in 50 patients with deep endometriosis, 4DryField^®^PH reduced the extent and severity of adhesions by 85%, and reduced adhesion incidence by 53%, relative to controls (Krämer *et al.*, 2021). At present, barrier agents may be considered in high-risk endometriosis surgery, particularly when extensive peritoneal stripping or ovarian surgery is performed.

### Use of ovarian suspension in endometriosis-related adhesion prevention

Ovarian suspension (ovariopexy) temporarily positions the ovary away from healing surfaces to reduce risk of adherence to injured mesothelium (Hoo *et al.*, 2014). In an RCT, ovarian suspension for 36–48 h post-laparoscopy failed to reduce adhesion development (Hoo *et al.*, 2014). Conversely, endometriosis-related laparoscopy with 36–48 h of ovarian suspension caused moderate adhesion formation in 18% of patients, versus 76% in the control group 3 months post-operatively (Hoo *et al.*, 2011). Furthermore, ovariopexy following endometriosis surgery reduced adhesion formation and improved dysmenorrhoea (Hoo *et al.*, 2011) and fertility (Ouahba *et al.*, 2004; Carbonnel *et al.*, 2011). For ovariopexy following endometriosis surgery, Seracchioli *et al.* (2014) used suture thread that readsorbed within 45–70 days, and suggested this timeframe would permit full peritoneal healing. Although evidence is conflicting, ovarian suspension may be considered in extensive endometriosis surgery involving adnexal dissection, particularly in fertility-preserving cases. Nevertheless, we must determine the optimum suture material and timeframe for ovarian suspension.

### Pharmacotherapy for endometriosis-related adhesion prevention

Pharmacological agents have been studied experimentally and/or clinically as post-operative adhesion-prevention strategies. Steroids had limited effectiveness at preventing adhesion formation in animal studies, and as they precipitate prolonged wound healing and immune suppression, they may be inappropriate following surgery (Arung *et al.*, 2011). Nevertheless, adhesion incidence has not been used as an outcome measure following steroid administration in gynaecological surgery (Ahmad *et al.*, 2020).

As downregulation of fibrinolysis promotes adhesion formation (Tabibian *et al.*, 2017), agents that increase peritoneal fibrinolytic capacity are attractive anti-adhesion options. Plasminogen activators streptokinase and urokinase were limited by their immunogenic action. Nevertheless, the second-generation plasminogen activator alteplase does not exhibit antigenic action, and its side effect profile in humans is well-established. However, research concerning its use in endometriosis surgery is lacking (Hellebrekers *et al.*, 2000, 2009).

### Effect of oestrogen modulation on preventing endometriosis-related adhesions

Hyperoestrogenism is a feature of endometriosis and may precipitate adhesiogenesis. Inducing a hypo-oestrogenic state using aromatase inhibitors or gonadotropin-releasing hormone (GnRH) agonists ameliorated adhesion formation in experimental models (Wright and Sharpe-Timms, 1995; Sharpe-Timms *et al.*, 1998). It has been demonstrated that preoperative GnRH agonist treatment, which induces a hypo-oestrogenic state, significantly reduces key inflammatory cytokines and growth factors in the peritoneal fluid of women with severe endometriosis, suggesting partial regression of the inflammatory peritoneal microenvironment (Nirgianakis *et al.*, 2016); however, a direct effect on the formation or prevention of adhesions has not yet been demonstrated. Furthermore, inhibiting 17β-hydroxysteroid dehydrogenase type 1, which catalyzes oestradiol production, reduced endometriotic lesions/adhesions in 60% of baboons with experimental endometriosis. However, sample sizes were small, and the study period was relatively short (Poirier *et al.*, 2022). In a mouse model of endometriosis, subcutaneous co-administration of oestradiol and progesterone for 4 weeks following ovariectomy reduced adhesion formation (Li *et al.*, 2016), likely due to antagonistic effects of progesterone on oestradiol. Progesterone administration reduced adhesion formation in animal models, but these results have not translated to humans (Montanino-Oliva *et al.*, 1996; Kaya *et al.*, 2007; Pados *et al.*, 2010). In endometriosis patients, previous coil use resulted in a 2.9-fold increased risk of adhesion formation (Forman *et al.*, 1993). The study did not clarify the coil type, yet a correlation should be investigated given the extensive use of the Mirena coil in endometriosis management (Kim *et al.*, 2022).

## Discussion

Understanding why endometriosis predisposes to the formation of peritoneal adhesions is of critical clinical importance, as adhesions contribute substantially to chronic pelvic pain, infertility, surgical complications and healthcare burden in affected patients. Despite their high prevalence, the biological mechanisms linking endometriosis to adhesiogenesis remains limited.

Evidence reviewed here supports a model in which adhesions in endometriosis arise from dysregulation of physiological peritoneal repair pathways. While coagulation, fibrinolytic imbalance, inflammation, hypoxia, and fibroproliferative signalling are recognized drivers of adhesiogenesis following surgical injury, endometriosis introduces persistent stimuli that may prevent resolution of these repair processes. Recurrent endometriotic lesion activity, bleeding, endocrine signalling, and immune dysregulation collectively sustain a thrombo-inflammatory microenvironment that favours fibrin persistence and fibrotic remodelling. Thus, rather than representing a distinct pathological entity, endometriosis-associated adhesiogenesis may reflect chronic activation of wound-healing mechanisms.

There are major unresolved questions concerning the cellular origins and functional specialization of myofibroblasts driving extracellular matrix deposition during adhesiogenesis. Current evidence suggests contributions from mesothelial-to-mesenchymal transition (MMT) and sub-mesothelial fibroblast activation (Foley-Comer *et al.*, 2002; Yáñez-Mó *et al.*, 2003; Chen *et al.*, 2014; Sandoval *et al.*, 2016; Uyama *et al.*, 2019; Foster *et al.*, 2020; Zindel *et al.*, 2021a), yet their relative importance remains uncertain and may vary depending on injury context. Determining whether myofibroblasts involved in endometriosis-associated adhesions differ from those participating in physiological peritoneal repair represents an important research question. Similarly, the source of peritoneal mesothelial cells involved in re-mesothelialization is unresolved, and understanding whether distant or circulating progenitors contribute to repair could inform regenerative therapeutic strategies (Zheng *et al.*, 2024).

Interactions between endometriotic lesions and surrounding peritoneum likely represent a critical mechanistic interface. Crosstalk between endometriotic cells and peritoneal mesothelial cells may alter fibrinolysis, enhance fibrinogenesis, or impair restoration of mesothelial integrity, thereby lowering the threshold for adhesiogenesis. Pro-fibrotic mediators such as TGF-β, which link endometriotic lesional fibrosis with adhesion biology, represent promising targets for interventions capable of simultaneously modifying endometriotic lesion progression and adhesiogenesis. Whether adhesions themselves subsequently influence endometriotic lesion establishment or persistence remains unknown and warrants investigation. Endocrine regulation also adds an additional driver to these processes, with oestrogen signalling promoting fibrogenic pathways in endometriosis and potentially in adhesions too. Determining whether hormonal therapies used in endometriosis management modify adhesion risk represents an important translational opportunity requiring epidemiological and mechanistic investigation.

Immune dysregulation provides an additional layer of complexity. Endometriosis is characterized by altered macrophage function, persistent inflammatory signalling juxtaposed with immune tolerance alongside platelet activation, all of which intersect with pathways governing peritoneal repair. Emerging evidence suggests that reparative immune responses may promote fibrosis when chronically active. Defining immune cell phenotypes and their temporal roles during adhesion and maturation will be essential for the development of targeted anti-adhesiogenic therapies that preserve normal peritoneal healing.

A significant barrier to progress in this field is the lack of suitable animal models that recapitulate adhesion formation in the setting of endometriosis, rather than from isolated surgical or ischemic injury. No studies have induced endometriosis in mice that model pathological peritoneal healing and peritoneal adhesions, and this should be a focus in the future. Clinically, the absence of non-invasive biomarkers and reliance on surgical diagnosis restrict longitudinal investigation of adhesion development. The adoption of a standardized scoring system, such as the peritoneal adhesion index (Coccolini *et al.*, 2013), would improve comparability across studies and accelerate translational progress.

Ultimately, prospective clinical studies are essential to elucidate the natural history of adhesions in endometriosis, including the prevalence, timing, and contribution to disease recurrence and surgical outcomes. Improved risk stratification may enable personalized surgical decision-making and inform preventative strategies, including optimization of surgical timing, refinement of operative techniques, and targeted pharmacological interventions.

## Conclusion

Peritoneal adhesions should be considered a central component of the endometriosis disease phenotype, rather than a secondary surgical complication alone. Current evidence suggests endometriosis alters the peritoneal environment in ways that sustain wound-healing responses and favour fibrotic remodelling, thereby increasing susceptibility to adhesiogenesis. Clarifying the mechanisms linking endometriotic lesion biology with pathological peritoneal repair will be essential for developing effective preventative and therapeutic strategies. Addressing this unmet clinical need has the potential to reduce pain, infertility, and surgical morbidity, ultimately improving quality of life for patients with endometriosis and reducing healthcare burden.

## Data Availability

No new data were generated or analyzed in support of this research.
